# Project Tycho 2.0: a repository to improve the integration and reuse of data for global population health

**DOI:** 10.1093/jamia/ocy123

**Published:** 2018-10-15

**Authors:** Willem G van Panhuis, Anne Cross, Donald S Burke

**Affiliations:** 1 Department of Epidemiology, University of Pittsburgh Graduate School of Public Health, Pittsburgh, Pennsylvania, USA; 2 Department of Biomedical Informatics, University of Pittsburgh School of Medicine, Pittsburgh, Pennsylvania, USA; 3 Public Health Dynamics Laboratory, University of Pittsburgh Graduate School of Public Health, Pennsylvania, USA

**Keywords:** global health, information storage and retrieval, public health surveillance, communicable diseases, information dissemination

## Abstract

**Objective:**

In 2013, we released Project Tycho, an open-access database comprising 3.6 million counts of infectious disease cases and deaths reported for over a century by public health surveillance in the United States. Our objective is to describe how Project Tycho version 1 (v1) data has been used to create new knowledge and technology and to present improvements made in the newly released version 2.0 (v2).

**Materials and Methods:**

We analyzed our user database and conducted online searches to analyze the use of Project Tycho v1 data. For v2, we added new US data and dengue data for other countries, and grouped data into 360 datasets, each with a digital object identifier and rich metadata. In addition, we used standard vocabularies to encode data where possible, improving compliance with FAIR (findable, accessible, interoperable, reusable) guiding principles for data management.

**Results:**

Since release, 3174 people have registered to use Project Tycho data, leading to 18 new peer-reviewed papers and 27 other creative works, such as conference papers, student theses, and software applications. Project Tycho v2 comprises 5.7 million counts of infectious diseases in the United States and of dengue-related conditions in 98 additional countries.

**Discussion:**

Project Tycho v2 contributes to improving FAIR compliance of global health data, but more work is needed to develop community-accepted standard representations for global health data.

**Conclusion:**

FAIR principles are a valuable guide for improving the integration and reuse of data in global health to improve disease control and save lives.

## BACKGROUND AND SIGNIFICANCE

Decisions in global population health can affect the lives of millions of people and can change the future of entire communities. For example, the decision to declare an influenza pandemic and stockpile vaccines can save millions of lives if a pandemic of highly pathogenic influenza actually occurred, or could waste millions of dollars if the decision was based on false alarm.^1^ Decision making in global health is often made under a high degree of uncertainty and with incomplete information. New data are rapidly emerging from mobile technology, electronic health records, and remote sensing.^2^ These new data can expand opportunities for data-driven decision making in global health. In reality, multiple layers of challenges, ranging from technical to ethical barriers, can limit the effective (re)use of data in global health.^3^^,^^4^ For example, composing an epidemic model to inform decisions about vaccine stockpiling requires the integration of existing data from a wide range of data sources, such as a population census, disease surveillance, environmental monitoring, and research studies.^5^ Integrating data can be a daunting task, especially since global health data are often stored in domain-specific data siloes that can each use different formats and content standards, ie, they can be syntactically and semantically heterogeneous. The heterogeneity of data in global health can slow down scientific progress, as researchers have to spend much time on data discovery and curation.^6^

To improve access to standardized data in global health, we created the Project Tycho data repository in 2013.^7^ The first version of Project Tycho (v1) comprised over a century of infectious disease surveillance data for the United States that had been published in weekly reports between 1888 and 2014.^7^ Weekly US disease surveillance reports were previously available in PDF and HTML formats on various online repositories and were not usable for research without substantial data curation. We digitized, transformed, and standardized these data and publicly released the entire database through www.tycho.pitt.edu. Since the public release of Project Tycho in 2013, over 3000 users have registered and have used Project Tycho data for research and technology development, leading to 45 new creative works. Now, we have released Project Tycho version 2.0 (v2). We have updated the content of Project Tycho data with new weekly US disease surveillance data, and we have added surveillance data for dengue-related conditions from 98 additional countries. In addition, we have redesigned the Project Tycho data format by grouping data into 360 datasets. Each dataset can now be identified by a digital object identifier (DOI) and can have its own metadata. We followed FAIR (findable, accessible, interoperable, and reusable) guiding principles where possible during the design of data and metadata representations.^8^ In this paper, we describe how others have used Project Tycho v1 data, illustrating the value of investing in a domain-specific open-data resource for accelerating science and creating new knowledge. We also describe the significant update of Project Tycho into version 2.0 with new data and improved FAIR compliance, towards a FAIR compliant data repository for global population health.

## METHODS

### Analyze Project Tycho data use

We analyzed our database of users who registered between the release of Project Tycho v1 (November 28, 2013) and December 31, 2017, to describe the type of users and the data they downloaded. To access Project Tycho data, users have to create an account with their name, institutional affiliation, country, and email. Users entered their information, except country, as free text. We standardized the names of the 100 most commonly listed institutions. We then classified users into 4 categories: 1) academic, 2) government, 3) personal, 4) other. We transformed all text to uppercase and classified all affiliations matching the regular expression “UNIVERSITY|UNIV|COLLEGE|SCHOOL|RESEARCH|FACULTY|ECOLE” as academic, affiliations matching the regular expression “NATIONAL|AGENCY|MINISTRY|^US|DEPARTMENT|DEPT” as government, affiliations with words “‘NONE’, ‘N/A’, ‘SELF’, ‘PERSONAL’, ‘PRIVATE’, ‘STUDENT’, ‘HOME’, ‘-’, ‘RETIRED’, ‘INDIVIDUAL’, ‘ME’, ‘INDEPENDENT’, ‘NA’, ‘NOT APPLICABLE’, and ‘CONSULTANT’” as personal, and all remaining affiliations as other. For all users first classified in the “other” category, we classified users with an email address ending with “.edu” as academic, with “gmail.com” as personal, with “.com” and not “gmail.com” as corporate, and with “.gov” as government. All users who could not be classified remained in the “other” category. Since November 28, 2013, we have recorded the queries and downloads made by users through the online Graphical User Interface (GUI). When signing up for an account, users agree to a privacy statement that notifies them of query and download tracking. Since February 4, 2014, we have also recorded calls to our Application Programming Interface (API). We extracted the disease names from user queries and analyzed the number of queries for each disease by user category.

We also conducted online searches to determine what creative works have resulted from use of Project Tycho v1 data by others (not including our own team). We defined a creative work as any publication (journal, newspaper, magazine, blog, website), algorithm, or software application that used Project Tycho data. During registration, users had to agree to an attribution license that requires citation of our primary paper describing the Project Tycho database, published in 2013 in the *New England Journal of Medicine*.^7^ We conducted searches in Google Scholar for citations of the primary Project Tycho paper to identify creative works potentially based on Project Tycho data. To identify papers that potentially used Project Tycho data, but did not cite the primary paper, we also searched PubMed, Scopus, Web of Science, Google Scholar, Google, Github, The Comprehensive R Archive Network (CRAN), Dryad, Figshare, and Zenodo for titles and/or abstracts containing the words “Project Tycho” and combinations of “Project Tycho” and disease names, or the words “vaccines” or “data.” We included only creative works published between the release of Project Tycho v1 and December 31, 2017. We then reviewed the main content of all papers and other creative works that potentially used Project Tycho data and verified that data used was indeed derived from Project Tycho by reviewing data source descriptions and references. All papers that cited the Project Tycho primary paper but that did not use Project Tycho data were categorized as citations only and were not included in the analysis of Project Tycho data use.

### Expanding data content

Project Tycho v1 included weekly National Notifiable Disease Surveillance System (NNDSS) reports published by the US Centers for Disease Control and Prevention (CDC) between September 9, 1887, and August 2, 2014. For version 2.0, we updated the US weekly surveillance data until the last week of 2017 by retrieving NNDSS data from the API of the CDC Morbidity and Mortality Weekly Report (MMWR).^9^ We standardized new NNDSS data into the Project Tycho standard format for pre-compiled datasets. We also added surveillance data for dengue-related conditions that we previously retrieved from the WHO DengueNet database, from WHO regional office websites, and from Ministries of Health in partner countries in Southeast Asia. We have previously published details about the origin and collection methods of these dengue data.^10^^,^^11^ Dengue surveillance data comprised counts of the number of cases or deaths reported for a specific location (country, first administrative subdivision, second administrative subdivision) through passive disease surveillance systems.

### Improved findability, accessibility, interoperability, and reusability

We grouped Project Tycho data into pre-compiled datasets in which each comprises all data from one condition in one country. Many users conduct disease- or country-specific analysis, making country-condition groups of data a natural fit. A dataset can include a large heterogeneity of information, eg, for a diversity of sublocations in a country and from numerous different sources. We also enabled the creation of custom datasets by users through a “create-your-own” GUI or the API.

Grouping data into pre-compiled datasets enabled us to assign a DOI to each dataset, and to create a standard metadata file for each dataset. We minted Project Tycho DOI’s consisting of a standard sequence “10.25337/T7/PTYCHO.V2.0/” and a unique sequence for each dataset. A dataset DOI resolves to the landing page of the dataset. Each landing page lists the dataset DOI, the download links for the data file (Comma Separated Value (CSV) format) and metadata files, and information about the condition, country, and time period represented by the dataset. We also listed the citation of the dataset on its landing page. For each dataset, we created metadata in the XML format specified by DataCite^12^ and in the JSON format specified by the Data Tag Suite (DATS).^13^

We defined a standard data format for pre-compiled Project Tycho datasets that includes 20 variables, and a format for custom compiled datasets that includes one additional variable, ie, the DOI of pre-compiled datasets from which each count was derived. Datasets are comprised of counts of cases or deaths due to certain conditions as reported by public health surveillance. The dataset variables represent attributes of counts, such as the location, time interval, condition, pathogen, etc. For each attribute, we tried to use a standard vocabulary or ontology. We searched the BioPortal and FAIRsharing catalogues, and the Google search engine to find appropriate standard vocabularies or ontologies.

We encoded metadata for each dataset according to the DataCite XML and DATS JSON schemas. We first compiled information for as many metadata attributes as possible. For most attributes, information could be derived from the dataset itself, but for some attributes we conducted additional online searches. For example, we conducted additional searches for contact information and ORCID identifiers for dataset creators and authors. We searched the BioPortal and FAIRsharing catalogues, and the Google search engine to find appropriate standard vocabularies or ontologies to represent metadata attributes. A valuable component of the DATS schema is the “CitedBy” attribute that enables representation of papers that have cited a dataset, in the dataset metadata. Although Project Tycho v1 data have been used by others, we did not use the “CitedBy” attributes yet because users have not yet cited the version 2.0 datasets and their DOIs. We plan to regularly update our dataset metadata with new “CitedBy” information, as users start to cite version 2.0 datasets.

## RESULTS

Project Tycho v1 comprised 3 666 141 provisional weekly counts of cases and deaths due to 50 infectious disease conditions (Supplementary Table S1), reported by the US NNDSS between September 9, 1887, and August 2, 2014. In the United States, the number of cases or deaths due to a select set of infectious diseases has been reported routinely for weekly time intervals since 1887 in public health journals such as *Public Health Reports* and MMWR. The US CDC and its precursors have published disease counts on a provisional basis every week and the final counts in annual summary reports.^14^ Project Tycho v1 included the provisional counts because the weekly time resolution enabled a greater range of epidemiological investigations compared to annual data, as illustrated by published work based on Project Tycho v1 data (https://www.tycho.pitt.edu/featured-works/).

### Project Tycho user community

**Figure 1.**
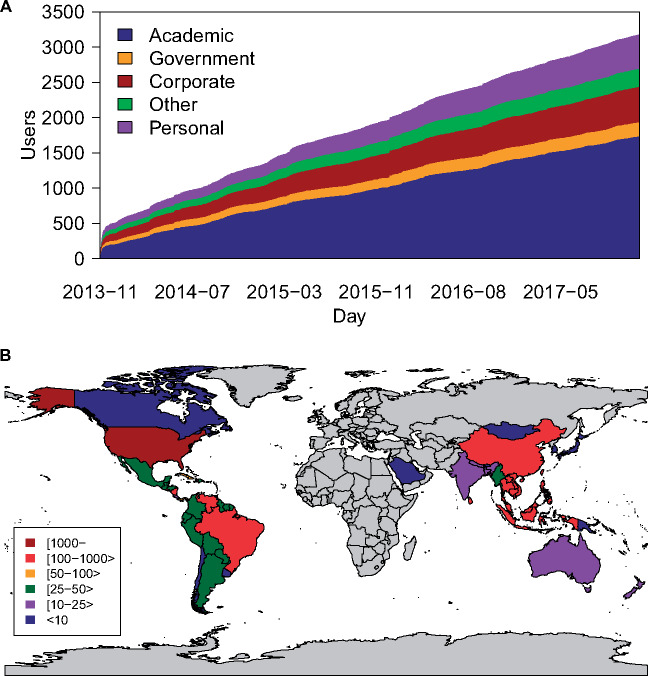
Project Tycho v1 registered users by day and country. (A) Number of user registrations per day since the release of Project Tycho v1, by type of user affiliation; (B) number of registered users per country.

Between November 28, 2013, and December 31, 2017, 3174 new users registered to access Project Tycho data (Figure 1A). We identified 1869 unique institutional affiliations. About one third (1203) of all registered users were affiliated with one of the 100 most frequently listed institutions (Supplementary Table S2). Over half of the users (1734) had an academic affiliation, 502 had a corporate affiliation (16%), 200 listed a government affiliation (6%), 479 listed no affiliation (15%), and for the 259 remaining users, we could not determine the type of affiliation (8%). Most users were based in the United States (2208), followed by the United Kingdom (133) and India (101). Users represented a total of 92 countries (Figure 1B).

### Data reuse and knowledge generation

**Figure 2.**
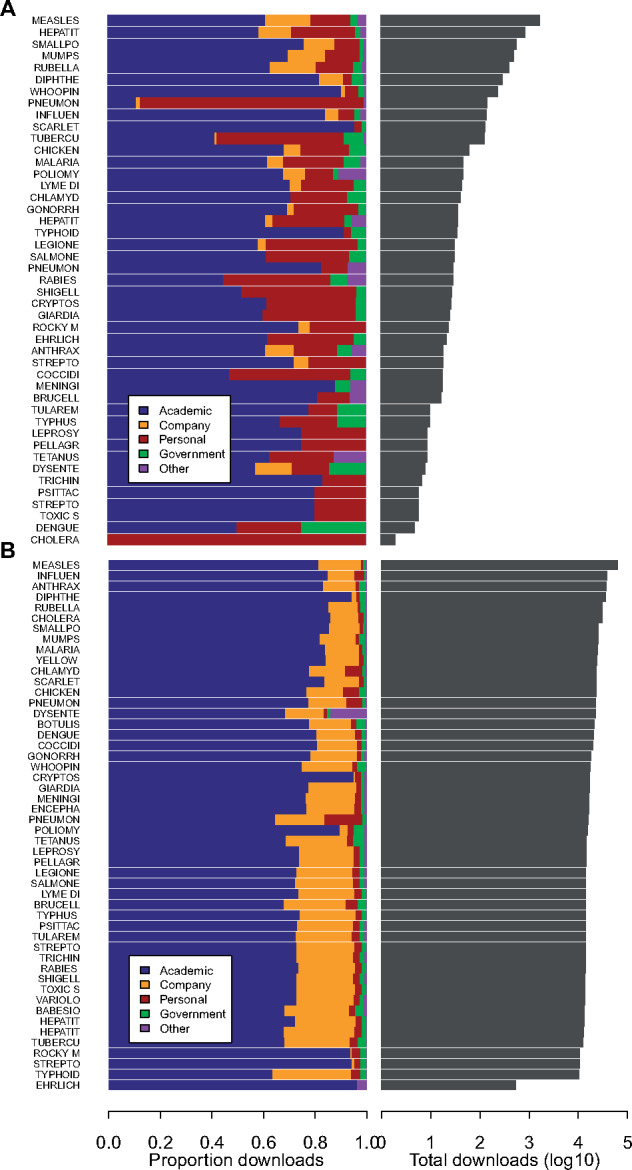
Project Tycho v1 data downloads by condition and type of user affiliation. (A) Proportion of datasets downloaded through the graphical user interface by each type of user affiliation, per condition, and the total number of datasets downloaded per condition in gray; (B) as (A), but for API calls.

**Figure 3.**
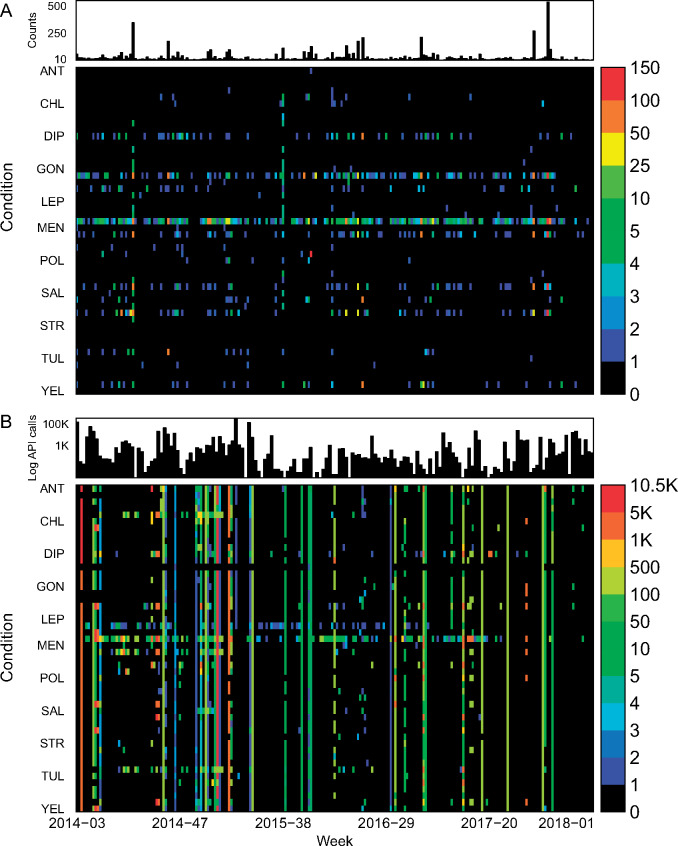
Project Tycho v1 datasets downloaded per condition and week. (A) Weekly number of datasets downloaded through the graphical user interface, per condition, with weekly totals in the top panel; (B) as (A), but for API calls.

Users could download Project Tycho v1 data through our online GUI or via calls to our API. Between release and December 31, 2017, 1048 distinct users have downloaded 6809 datasets through our GUI. We started tracking API calls on February 4, 2014, and between then and December 31, 2017, 161 users made 1 022 480 API calls. Seventy-four users downloaded data through both the GUI and the API. The maximum number of API calls made by one user was 390 519, and the maximum number of datasets downloaded by one user through the GUI was 375. We found that 6757 GUI downloads and 953 712 API calls included disease-specific information. We explored the number of downloads per disease and user affiliation (Figure 2). GUI users downloaded the most datasets for measles (1682), followed by Hepatitis A (851) and Pertussis (753) (Figure 2A). The distribution of conditions selected for downloading was not identical among the different types of users. For example, all cholera datasets were downloaded by personal users (not affiliated to a government, academic, or corporate institution), and a larger percentage of dengue datasets were downloaded by government users compared to other conditions (Figure 2A). User preferences for specific datasets likely reflected a combination of data availability and user interest. For example, the longest time series are available for measles and pertussis, and these diseases are also very highly studied in the infectious disease epidemiology domain; and the emergence of dengue is likely a concern for government health agencies. The relatively large proportion of datasets downloaded by personal users likely reflects a high level of interest among the general public in infectious diseases such as cholera and pneumonia, although these data should be interpreted carefully, as many professional users may not have listed their affiliation or may have used their Gmail address to login. We inspected the distribution of diseases downloaded by users across countries and did find very similar patterns across countries, suggesting no relationship between the disease burden in user countries and user interest in specific diseases. Users from India downloaded relatively more data on respiratory diseases compared to users from other countries, possibly related to the importance of respiratory diseases in India. Disease preferences were slightly different for API users (Figure 2B). API calls most frequently included measles (63 589), followed by influenza (38 460), and anthrax (37 205). Interestingly, API calls were almost exclusively made by academic and corporate users. It is likely that these user groups have more advanced computational capability enabling them to make API calls compared to government or personal users. Project Tycho data for anthrax are very limited, which is immediately visible through the GUI, but not before an API call is made, explaining why GUI users did not show the same interest in anthrax as API users. We also studied the data download patterns over time (Figure 3). Data for most conditions were downloaded continuously throughout the time period, but behavior was different for the API vs the GUI. API calls included all conditions much more frequently compared to the GUI, reflecting the advantage of the API vs the GUI for accessing data for multiple conditions at once, which would be much more laborious through the GUI.

**Figure 4.**
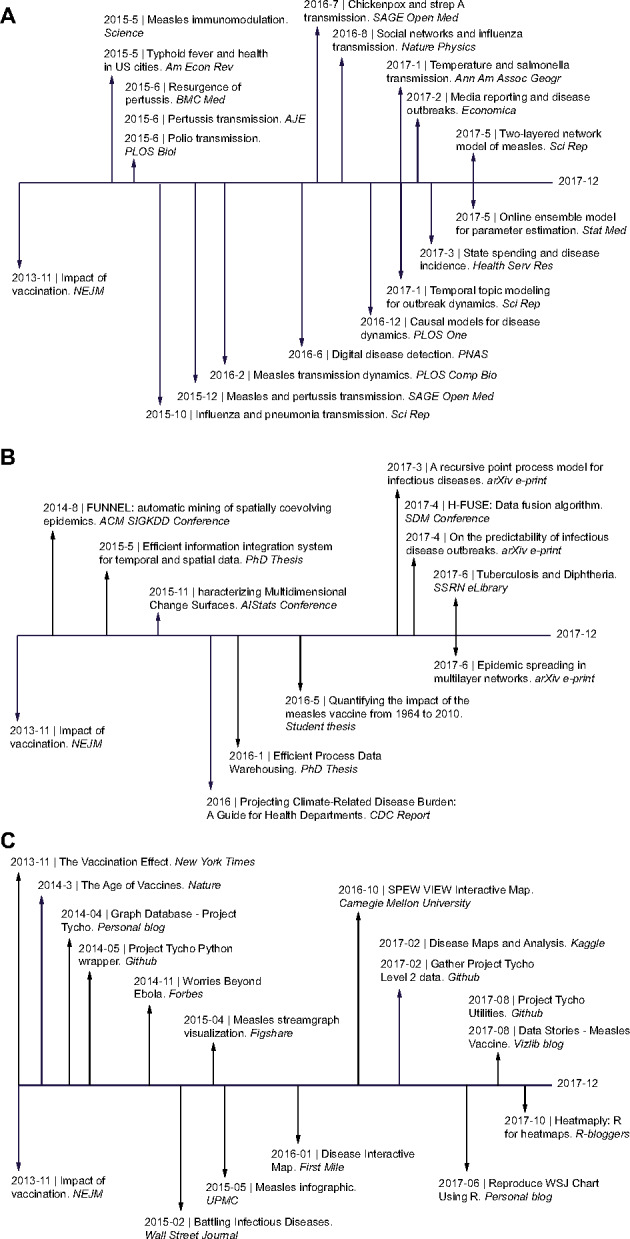
Creative works resulting from Project Tycho v1 data. (A) Peer-reviewed papers published that used Project Tycho v1 data in chronological order; (B) as (A), but for conference papers, pre-prints, and student theses; (C) as (A), but for newspaper articles, visualizations, and software.

We identified new knowledge generated based on Project Tycho data. We found 150 published works that cited the Project Tycho release paper, 47 published by authors from one of the 100 institutions most commonly listed as affiliation by registered Project Tycho users. Not all works that cited the primary Project Tycho paper used our data to generate new knowledge. We found 45 creative works published before December 31, 2017, including 18 peer-reviewed papers, 8 conference papers or pre-prints, 3 student theses, and 16 newspaper, blog, or website articles that used Project Tycho data and that were not produced by our own team (Figure 4, Supplementary Table S3). Based on the acknowledgments made in published papers, we found that 5 papers were funded by a foundation, 6 papers were funded by the US National Institutes of Health, 6 papers by the United States or China National Science Foundation, and 1 paper was funded by industry grants. Most of the new knowledge derived from Project Tycho data was about disease transmission patterns for respiratory pathogens (measles, pertussis, chickenpox, streptococcus) and fecal-orally transmitted pathogens (polio, hepatitis A, typhoid). Project Tycho data were also used to create new technology including statistical clustering, machine learning, data mining, disease forecasting algorithms, and visualization software. New knowledge or technology was not only created by post-doctoral researchers, but also by pre-doctoral students, including a master’s thesis on measles vaccination in the United States and 2 doctoral theses describing new data integration methods. Furthermore, Project Tycho data were used for advocacy, eg, by news articles about the importance of vaccination programs and disease surveillance.

Project Tycho v1 data have also been used by training programs. For example, the University of Pittsburgh organized an Undergraduate Data Palooza in 2012 and 2013, for which undergraduate students from around the world used Project Tycho data to study historical disease patterns. The three best submissions won a $1000 prize each. The Last Mile initiative used Project Tycho data to train inmates in the 2015 class of the San Quentin 7370 program for web development. The 2017 Computational Biology of Infectious Disease (CBID) course in Southeast Asia, organized by the French Institute of Research for Development (IRD), used Project Tycho data to train students in statistical analysis of disease surveillance data. Examples of using Project Tycho data for training programs became known to us due to our personal involvement or because colleagues mentioned it to us. More examples could exist that have remained unknown to us.

### Project Tycho version 2.0

**Figure 5.**
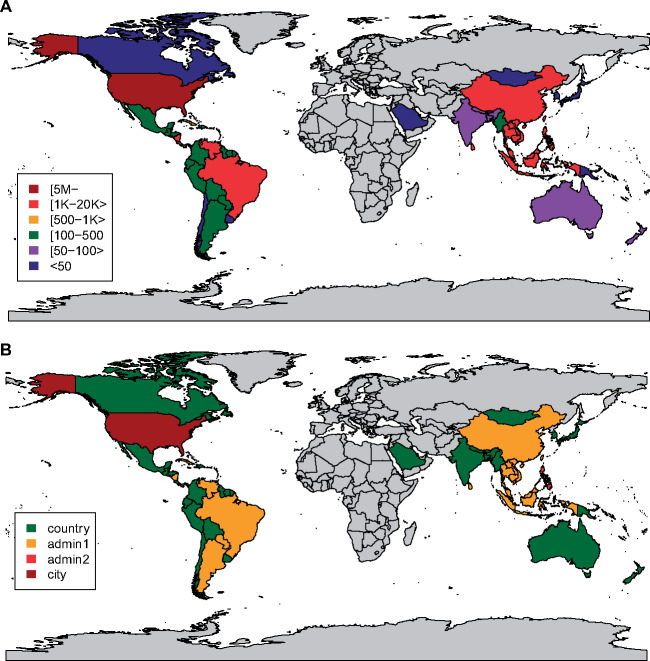
Project Tycho v2 data available per country. (A) Number of counts available for each country; (B) highest spatial resolution of data available for each country.

**Figure 6.**
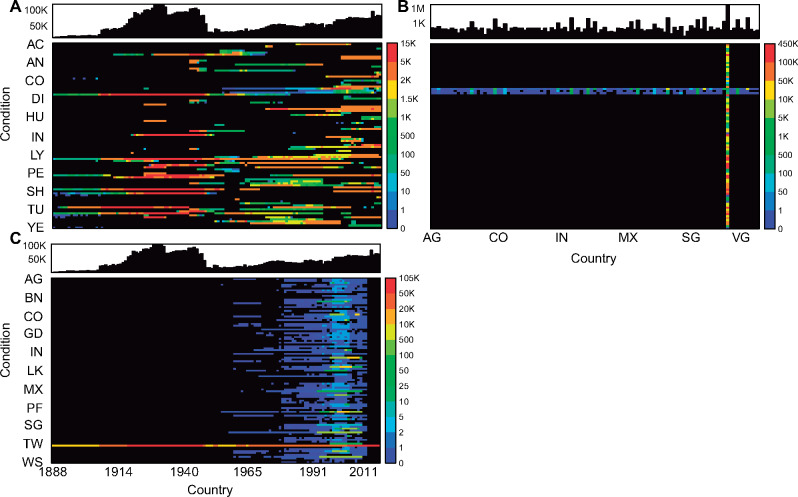
Project Tycho v2 data available per condition, country, and year. (A) Annual number of counts available for each condition (ranked alphabetically), with yearly totals in the top panel; (B) number of counts available for each condition and country (both ranked alphabetically), with country totals in the top panel; (C) annual number of counts available for each country (ranked alphabetically), with annual totals in the top panel.

For Project Tycho v2, we updated the US data and added dengue data for 98 additional countries. We added new US NNDSS counts published in weekly MMWR surveillance tables between August 2, 2014, and December 31, 2017. We compiled dengue data from 13 different sources including the World Health Organization, and from country-specific databases from 8 countries in Southeast Asia (Supplementary Table S4), as previously described.^10^^,^^11^ We also improved the data format, standardization, and metadata, following FAIR guiding principles where possible. Project Tycho v2 includes 5 676 424 counts of cases or deaths due to reported disease conditions, an increase of 50% from v1. Version 2.0 data represent 99 countries, 92 conditions, and 58 pathogens (Supplementary Table S5). We could not assign a pathogen to each condition because some conditions were not caused by a pathogen, such as aseptic meningitis, and some conditions could be caused by pathogens from multiple superkingdoms, such as dysentery. We grouped data into pre-compiled datasets in which each comprises all counts for a single condition in one country. For example, we grouped all counts for measles in the United States in one dataset. We created 360 datasets. Ninety-two datasets are for disease conditions in the United States, and 268 are for up to 3 dengue-related conditions in other countries (Figure 5). Datasets for conditions in the United States can include counts reported between 1887 and 2017, while most datasets for dengue-related conditions in other countries include counts reported between 1960 and 2012 (Figure 6).

### Findability

We created a DOI for each Project Tycho v2 pre-compiled dataset. Users can also compile custom datasets through the online GUI or API, but custom datasets do not have DOIs because there are an infinite number of possible datasets that can be compiled by users. For example, Project Tycho v1 users have created over 300 000 datasets in 1 day through the API. For custom-compiled datasets, we include, for every count, the DOI of the pre-compiled dataset that also includes that count. The DOIs will enable users to cite each dataset and will enable us to more accurately detect the reuse of datasets by others. We also created metadata for each dataset in XML format following the DataCite schema,^12^ and in JSON format following the DATS schema developed by the Biomedical and Healthcare Data Discovery Index Ecosystem (bioCADDIE).^13^ We used metadata attributes and values from standard vocabularies and ontologies where possible. We used 82 attributes from the DataCite XML schema to describe each Project Tycho dataset (Supplementary Table S6), and 163 attributes from the DATS specification (Supplementary Table S7). The main attributes, included in both schemas, were the dataset identifier, creators, subjects (IsAbout attribute in DATS), license, associated publications, and funders. In addition, the DATS schema included information about the distribution of the dataset (CSV files) and about the Project Tycho repository containing the distributions. We used the DataCite XML metadata files to mint DOIs for our datasets through the University of California EZID service.^15^ Project Tycho 2.0 datasets are now indexed by the EZID catalogue at the University of California and by DataCite.^16^ We also registered Project Tycho with the DataMed catalogue for indexing.^17^ Through our participation in the Models of Infectious Disease Agent Study (MIDAS), Project Tycho 2.0 datasets have also been indexed by the MIDAS Digital Commons (MDC).^18^ Furthermore, the Project Tycho database is registered with FAIRSharing^19^ and with various university libraries.

### Accessibility

Project Tycho pre-compiled datasets are listed on our website and can be accessed through download links on the dataset landing pages (Supplementary Figure S1A-B). The dataset landing pages list the DOI, the reported conditions and pathogens included in the dataset, the country included, and the time period covered by the dataset. Each landing page also includes a download link for metadata files. Users can also compile a custom dataset through the online GUI or API (Supplementary Figure S1C). For example, users may want to combine data from multiple conditions or countries in one dataset instead of downloading a pre-compiled dataset for each country-condition combination. Also, the GUI enables users to visualize data before downloading. Only authorized users can download data after authentication via their login or API web key. All users that have created a user account are authorized to download Project Tycho data. We have included the URL of the main data access page (www.tycho.pitt.edu/data) and of dataset-specific landing pages in dataset metadata files. We also encoded data access, authorization, and authentication procedures in the metadata.

### Interoperability

We have designed a standard Project Tycho data format for pre-compiled and for custom-created datasets and registered both formats with FAIRsharing (Supplementary Table S8).^19^ We used 17 standard vocabularies and ontologies to represent information in Project Tycho datasets (Table 1). All Project Tycho data are represented as counts of cases or deaths related to disease conditions reported by public health surveillance. We used Systematized Nomenclature of Medicine – Clinical Terms (SNOMED-CT) names and codes to represent disease conditions, NCBI Taxonomy names and identifiers to represent pathogens, ISO-3166 names and codes to represent countries and first level administrative subdivisions, and names from the Geonames vocabulary to represent second-level administrative subdivisions and cities. We also used SNOMED-CT terminology to represent the reporting of cases or deaths due to diseases, and to represent diagnostic certainty. We did not use the International Classification of Diseases (ICD) vocabulary to encode disease conditions because many conditions were not specified in the ICD standard, and the SNOMED-CT ontology represented the logical relationships between conditions in more detail. If needed, existing maps between SNOMED and ICD vocabularies can be used to convert between the two.^20^ In metadata files, we also encoded information that applied to the entire dataset, including SNOMED-CT condition names and codes, NCBI Taxonomy names and identifiers for pathogens, and ISO 3166 country names and codes. In addition, we encoded information about the general type of data (disease surveillance), and the general method used to collect the data (disease notification) in the metadata. Also, we encoded the time period represented, identifiers and roles of researchers involved in creating datasets, and funding agencies involved (Supplementary Tables S6–S7).

**Table 1.** Standard vocabularies and ontologies used by Project Tycho data and metadata

Attribute	Vocabulary/ontology	Vocabulary/ontology identifier
Condition, case, death, diagnosis certainty	SNOMED-CT	doi: 10.25504/fairsharing.d88s6e
Country and Admin1	ISO-3166	https://www.iso.org/standard/63545.html
Admin2 and city	Geonames	http://www.geonames.org/
Pathogen	NCBI Taxonomy	doi: 10.25504/fairsharing.fj07xj
Funding agency	Crossref Funder Registry	https://www.crossref.org/services/funder-registry/
Fatality	SNOMED-CT	doi: 10.25504/fairsharing.d88s6e
Time intervals	Time Intervals Ontology	http://reference.data.gov.uk/def/intervals
Researchers	Open Researcher and Contributor ID (ORCID)	doi: 10.25504/fairsharing.nx58jg
Surveillance data	Apollo SV	doi: 10.25504/fairsharing.ngv2xx
Disease notification	Medical Subject Headings (MeSH)	doi: 10.25504/fairsharing.qnkw45
Contributor type, related identifier relation type	DataCite XSD	doi: 10.25504/fairsharing.me4qwe
Cumulative incidence	Epidemiology Ontology	http://www.ontobee.org/ontology/EPO
Dates	ISO 8061	https://www.iso.org/iso-8601-date-and-time-format.html
Infectious disease incidence	Infectious Disease Ontology	doi: 10.25504/fairsharing.aae3v6
Creator and author roles	Scholarly Contributions and Roles Ontology	http://purl.org/spar/scoro
Type of standard for dataset distribution	EMBRACE Data and Methods Ontology	doi: 10.25504/fairsharing.a6r7zs
Megabites	Unit Ontology	doi: 10.25504/fairsharing.mjnypw

### Reusability

We aimed to create rich metadata for pre-compiled Project Tycho datasets by using as many attributes as possible from the DataCite and DATS schemas (Supplementary Tables S6–S7). All Project Tycho data are licensed under a Creative Commons Attribution 4.0 International license that allows any use free of charge as long as Project Tycho is attributed as the source of the data.^21^ We list the dataset citation, that includes the DOI, on the landing page of each dataset to enable appropriate attribution (Supplementary Figure S1B). Users who compiled a custom dataset through the GUI or API can cite the DOI of each dataset from which counts for the custom dataset were taken.

## DISCUSSION

In this paper, we presented the value of sharing historical epidemiological data for creating new knowledge and technology with the example of Project Tycho v1 and improvements made for Project Tycho v2. We anticipate that the improved FAIR representation of Project Tycho (meta)data will lead to a broader and more efficient reuse of disease surveillance data for research and technology development. Our aim is to grow Project Tycho into a domain repository for global population health, serving as an integrated data resource for the global health community.

Creating an open-access repository for standardized information that was previously available only in PDF format required significant upfront investments of time and effort, but will be cost effective in the long run by avoiding duplication of data digitization and curation for many individual research projects. From a researcher perspective, digitizing and standardizing all data were more expensive than digitizing one specific section of interest. From a funder and community perspective, digitizing and standardizing all data in one sweep were more efficient and cost effective compared to doing the same work for small parts of the data at a time, as many operations are done only once, such as compiling all files, establishing standard operating procedures, opening, closing, and saving files, and standardizing file formats and contents. Updating Project Tycho v1 to v2 required an additional investment (50% faculty, 50% senior developer, and 100% junior developer full-time equivalent over the course of 5 months) for redesigning the data format, improving FAIR compliance, and for development of a new online user interface. The return on this investment will be further prevention of duplication and the acceleration of new science and discovery in global health through improved FAIR compliance of the Project Tycho data repository.

We presented new knowledge based on Project Tycho data in terms of published papers, theses, software products, etc., and expect that this knowledge will have a positive impact on global health. This impact will take time and will be difficult to measure. Project Tycho v1 has enabled research in 3 ways. First, the large spatial (entire United States) and temporal (1888-2013) scope has enabled studies to expand their scope and make more robust inference.^22–25^ For example, a highly impactful study on the immunomodulatory effect of measles infection, published by Mina et al. in *Science*,^22^ used US data from Project Tycho in conjunction with data from Denmark and the United Kingdom, expanding the scope of this study beyond Europe. Second, the standard format of Project Tycho data has enabled the application of new analytical algorithms developed by computer scientists to biomedical data,^23^^,^^26–29^ bridging the disciplinary gap between these domains. Without standardized data from Project Tycho, these scientists may have used non-medical data instead, possibly depriving the biomedical domain of new innovations. Third, the user-friendly format of Project Tycho data has led to the use of biomedical data by journalists for advocacy about the value of vaccination and surveillance in widely read magazines and newspapers,^30–33^ such as *Nature*,^30^ the *New York Times*,^31^ the *Wall Street Journal*,^32^ and *Forbes Magazine*,^33^ leading to a better informed general public. In many of these instances, the research would have been possible without Project Tycho, as demonstrated by similar papers that did not use Project Tycho data,^34–37^ but would have required significant investments in data digitization and standardization, thus duplicating efforts without resulting in an open-access, community resource.

With Project Tycho v2, we have contributed to improving FAIR compliance of data in global health, but more work remains to be done. For example, we used a set of 17 external standard vocabularies and ontologies to represent disease surveillance data, but many additional standards exist and may be applicable. It would be relevant for the global health community to develop a collection of preferred, existing standards and ontologies that can be used by most stakeholders. In addition, new standards and ontologies could be created for data attributes that cannot easily be represented with current standards. For example, it would be valuable to represent data provenance in detail, given the heterogeneity in data methods, sources, and workflows used in global health. Also, global health data are dynamic and always changing, which complicates data identifiability. Fortunately, metadata schemas have started to incorporate attributes that can represent changing data, eg, through the “relationType” attribute of the DataCite XML schema that can take on values such as “IsContinuedBy,” “Continues,” or “ISNewVersionOf” to represent relationships between datasets.^12^

## CONCLUSION

The FAIR principles are a valuable guide for improving the integration and reuse of global health data. Adoption of these principles by Project Tycho and other data repositories in global health can accelerate the integration and reuse of data to discover new knowledge and technology for improving the lives of populations around the world.

## FUNDING

This research was funded by the National Institute of General Medical Sciences Models of Infectious Disease Agent Study (MIDAS, 5U54GM088491-09), the NIH Big Data to Knowledge (BD2K) program (5K01ES026836-03 and 3K01ES026836-02S1), and the Bill and Melinda Gates Foundation (49276).

## CONTRIBUTOR

WGVP conceptualized the project, conducted the analyses, and wrote the manuscript; AC conducted the analysis and contributed to writing the manuscript; DSB conceptualized the project and contributed to the analysis and writing of the manuscript.

## SUPPLEMENTARY MATERIAL

Supplementary material is available at *Journal of the American Medical Informatics Association* online.

*Conflict of interest statement*. The authors declare no competing interests.

## Supplementary Material

Supplementary Data
