# Supplementary material for: Project Tycho 2.0: a repository to improve the integration and reuse of data for global population health
Source: J Am Med Inform Assoc. 2018 Oct 15;25(12):1608–17. doi: 10.1093/jamia/ocy123 (PMC6289551; doi:10.1093/jamia/ocy123)
Supplement: Supplementary Data [file ocy123_supp.docx]

**Project Tycho 2.0: A repository to improve the integration and reuse of data for global population health**

**Supplement**

Content

**Supplementary Tables**

- Supplementary Table 1, Conditions available in Project Tycho version 1

- Supplementary Table 2, Top 100 most listed affiliations for Project Tycho v1 users

- Supplementary Table 3, Creative works based on Project Tycho data

- Supplementary Table 4, Sources included in Project Tycho 2.0

- Supplementary Table 5, Conditions and pathogens included in Project Tycho 2.0

- Supplementary Table 6, DataCite attributes used in Project Tycho 2.0 metadata

- Supplementary Table 7, DATS attributes used in Project Tycho 2.0 metadata

- Supplementary Table 8, Project Tycho standard data format for pre-compiled datasets

**Supplementary Figures**

- Supplementary Figure 1, Screenshots of Project Tycho v2 data retrieval pages. (A) Listing of Project Tycho v2 pre-complied datasets; (B) the landing page of the pre-compiled dataset with counts of Rocky Mountain Spotted Fever in the United States; and (C) the graphical user interface for compiling a custom datasets

**References**

Supplementary Table 1, Conditions available in Project Tycho version 1

| **Condition** | **Subcategory** | **Counts** |
| --- | --- | --- |
| ANTHRAX | UNSPECIFIED | 7051 |
| BABESIOSIS | UNSPECIFIED | 36 |
| BOTULISM | UNSPECIFIED | 10 |
| BRUCELLOSIS [UNDULANT FEVER] | UNSPECIFIED | 14970 |
| CHICKENPOX [VARICELLA] | UNSPECIFIED | 75201 |
| CHLAMYDIA | UNSPECIFIED | 16837 |
| CHOLERA | UNSPECIFIED | 125 |
| COCCIDIOIDOMYCOSIS | UNSPECIFIED | 1069 |
| CRYPTOSPORIDIOSIS | UNSPECIFIED | 7590 |
| DENGUE | UNSPECIFIED | 623 |
| DIPHTHERIA | UNSPECIFIED | 380654 |
| DYSENTERY | UNSPECIFIED | 10493 |
| EHRLICHIOSIS/ANAPLASMOSIS | UNSPECIFIED | 136 |
| ENCEPHALITIS | UNSPECIFIED | 5 |
| GIARDIASIS | UNSPECIFIED | 10206 |
| GONORRHEA | UNSPECIFIED | 23840 |
| HEPATITIS A | ACUTE TYPE A | 51527 |
| HEPATITIS B | ACUTE TYPE B | 50452 |
| INFLUENZA | UNSPECIFIED | 236821 |
| LEGIONELLOSIS | UNSPECIFIED | 6692 |
| LEPROSY | UNSPECIFIED | 7677 |
| LYME DISEASE | UNSPECIFIED | 4706 |
| MALARIA | UNSPECIFIED | 12286 |
| MEASLES | UNSPECIFIED | 357229 |
| MENINGITIS | UNSPECIFIED | 29926 |
| MUMPS | UNSPECIFIED | 88899 |
| PELLAGRA | UNSPECIFIED | 33246 |
| PNEUMONIA | UNSPECIFIED | 214411 |
| PNEUMONIA AND INFLUENZA | UNSPECIFIED | 239793 |
| POLIOMYELITIS | TOTAL | 140807 |
| PSITTACOSIS | UNSPECIFIED | 824 |
| RABIES IN ANIMALS | UNSPECIFIED | 30755 |
| ROCKY MOUNTAIN SPOTTED FEVER | UNSPECIFIED | 18657 |
| RUBELLA | UNSPECIFIED | 26228 |
| SALMONELLOSIS | UNSPECIFIED | 12891 |
| SCARLET FEVER | UNSPECIFIED | 345640 |
| SHIGELLOSIS | UNSPECIFIED | 8673 |
| SMALLPOX | UNSPECIFIED | 275547 |
| STREPTOCOCCAL DISEASE, INVASIVE GROUP A | UNSPECIFIED | 3311 |
| STREPTOCOCCAL SORE THROAT | UNSPECIFIED | 4643 |
| TETANUS | UNSPECIFIED | 1913 |
| TOXIC SHOCK SYNDROME | UNSPECIFIED | 1256 |
| TRICHINIASIS | UNSPECIFIED | 737 |
| TUBERCULOSIS [PHTHISIS PULMONALIS] | UNSPECIFIED | 348296 |
| TULAREMIA | UNSPECIFIED | 15984 |
| TYPHOID FEVER [ENTERIC FEVER] | UNSPECIFIED | 305266 |
| TYPHUS FEVER | UNSPECIFIED | 11870 |
| VARIOLOID | UNSPECIFIED | 21 |
| WHOOPING COUGH [PERTUSSIS] | UNSPECIFIED | 230236 |
| YELLOW FEVER | UNSPECIFIED | 75 |
| **TOTAL** |  | **3666141** |

Supplementary Table 2, Top 100 most listed affiliations for Project Tycho v1 users

| **Institution** | **Number of users** |
| --- | --- |
| UNIVERSITY OF PITTSBURGH | 170 |
| NONE | 150 |
| UNIVERSITY OF CALIFORNIA | 116 |
| JOHNS HOPKINS UNIVERSITY | 43 |
| COLUMBIA UNIVERSITY | 31 |
| UNIVERSITY OF MICHIGAN | 26 |
| PENNSYLVANIA STATE UNIVERSITY | 25 |
| PRINCETON UNIVERSITY | 25 |
| UNIVERSITY OF FLORIDA | 25 |
| CARNEGIE MELLON UNIVERSITY | 23 |
| UNIVERSITY OF WASHINGTON | 21 |
| HARVARD UNIVERSITY | 20 |
| US CENTERS FOR DISEASE CONTROL | 19 |
| UNIVERSITY OF GEORGIA | 18 |
| UNIVERSITY OF VERMONT | 16 |
| VIRGINIA TECH | 15 |
| NEW YORK UNIVERSITY | 14 |
| OXFORD UNIVERSITY | 14 |
| UNIVERSITY OF MINNESOTA | 14 |
| SAS INSTITUTE | 13 |
| UNIVERSITY OF CHICAGO | 13 |
| IMPERIAL COLLEGE LONDON | 12 |
| MIT | 11 |
| YALE UNIVERSITY | 11 |
| BOSTON UNIVERSITY | 10 |
| GEORGE MASON UNIVERSITY | 10 |
| MINISTRY OF HEALTH | 9 |
| STANFORD UNIVERSITY | 9 |
| THE OHIO STATE UNIVERSITY | 9 |
| EMORY UNIVERSITY | 8 |
| GEORGETOWN UNIVERSITY | 8 |
| UNIVERSITY OF ARIZONA | 8 |
| UNIVERSITY OF WARWICK | 8 |
| US NATIONAL INSTITUTES OF HEALTH | 8 |
| METABIOTA | 7 |
| MICHIGAN STATE UNIVERSITY | 7 |
| UNIVERSITY OF MARYLAND | 7 |
| UNIVERSITY OF NOTRE DAME | 7 |
| UNIVERSITY OF PENNSYLVANIA | 7 |
| ARIZONA STATE UNIVERSITY | 6 |
| CAMBRIDGE UNIVERSITY | 6 |
| DUKE UNIVERSITY | 6 |
| LONDON SCHOOL OF HYGIENE AND TROPICAL MEDICINE | 6 |
| WORLD HEALTH ORGANIZATION | 6 |
| INSTITUT PASTEUR | 5 |
| LOS ALAMOS NATIONAL LABORATORY | 5 |
| MCMASTER UNIVERSITY | 5 |
| NORTHWESTERN UNIVERSITY | 5 |
| PACIFIC LUTHERAN UNIVERSITY | 5 |
| QUEEN'S UNIVERSITY | 5 |
| RUTGERS UNIVERSITY | 5 |
| TSINGHUA UNIVERSITY | 5 |
| UNIVERSITY OF SAO PAULO | 5 |
| UNIVERSITY OF SOUTH FLORIDA | 5 |
| UNIVERSITY OF TORONTO | 5 |
| BOOZ ALLEN HAMILTON | 4 |
| CARLETON COLLEGE | 4 |
| FLORIDA STATE UNIVERSITY | 4 |
| GSK | 4 |
| INSTITUTE OF NOTRE DAME | 4 |
| KENNESAW STATE UNIVERSITY | 4 |
| MYLAN | 4 |
| TEMPLE UNIVERSITY | 4 |
| TULANE UNIVERSITY | 4 |
| UNIVERSITY OF CALGARY | 4 |
| UNIVERSITY OF KENTUCKY | 4 |
| UNIVERSITY OF LIVERPOOL | 4 |
| UNIVERSITY OF SOUTHERN MAINE | 4 |
| UNIVERSITY OF VIRGINIA | 4 |
| UPMC | 4 |
| VANDERBILT | 4 |
| BOSTON CHILDREN'S HOSPITAL | 3 |
| BRIGHAM YOUNG UNIVERSITY | 3 |
| CALIFORNIA DEPARTMENT OF PUBLIC HEALTH | 3 |
| CLARK UNIVERSITY | 3 |
| COMMACK HIGH SCHOOL | 3 |
| CORNELL UNIVERSITY | 3 |
| DARTMOUTH COLLEGE | 3 |
| DENISON UNIVERSITY | 3 |
| DEPARTMENT OF HEALTH | 3 |
| DREXEL UNIVERSITY | 3 |
| FLORIDA DEPARTMENT OF HEALTH | 3 |
| FRED HUTCHINSON CANCER RESEARCH CENTER | 3 |
| GENERAL ASSEMBLY | 3 |
| GEORGIA INSTITUTE OF TECHNOLOGY | 3 |
| IOWA STATE UNIVERSITY | 3 |
| ISI FOUNDATION | 3 |
| MASSACHUSETTS GENERAL HOSPITAL | 3 |
| MIAMI UNIVERSITY | 3 |
| MICROSOFT | 3 |
| MIDLAND COLLEGE | 3 |
| NORTHEASTERN UNIVERSITY | 3 |
| NYU | 3 |
| OHIO STATE UNIVERSITY | 3 |
| OKLAHOMA STATE UNIVERSITY | 3 |
| PNNL | 3 |
| PWC | 3 |
| RICE UNIVERSITY | 3 |
| SAN DIEGO STATE UNIVERSITY | 3 |
| SHARE INDIA | 3 |
| **TOTAL** | **1203** |

Supplementary Table 3, Creative works based on Project Tycho data

| **Title** | **Funder*** | **Citation** |
| --- | --- | --- |
| **Peer-reviewed papers (18)** | | |
| The historical association between measles and pertussis: A case of immune suppression? |  | Coleman S. 2015[1] |
| The association between varicella (chickenpox) and group A streptococcus infections in historical perspective. |  | Coleman S. 2016[2] |
| Persistent Chaos of Measles Epidemics in the Prevaccination United States Caused by a Small Change in Seasonal Transmission Patterns. |  | Dalziel BD., et. al. 2016[3] |
| The role of influenza in the epidemiology of pneumonia. | NIGMS | Shrestha S., et. al. 2015[4] |
| Asymptomatic transmission and the resurgence of Bordetella pertussis. |  | Althouse BM., et al. 2015[5] |
| Long-term measles-induced immunomodulation increases overall childhood infectious disease mortality. | Homeland Security, BMGF, Fogarty | Mina MJ., et. al. 2015[6] |
| Dynamics of Pertussis Transmission in the United States. | NIGMS, NIAID | Magpantay FMG., et. al. 2015[7] |
| Unraveling the Transmission Ecology of Polio. | NIGMS, NIAID, NCRR, Fogarty | Martinez-Bakker M., et. al. 2015[8] |
| The effect of a prudent adaptive behaviour on disease transmission. | Santa Fe Institute, Omidyar Group, Fonds de recherche Quebec, James S. McDonnell Foundation | Scarpino SV., et. al. 2016[9] |
| Declining Mortality Inequality within Cities during the Health Transition. |  | Costa DL., et. al. 2015[10] |
| Synchronized and mixed outbreaks of coupled recurrent epidemics. | China Nat. Natural SF, China Nat. Basic Res prog, Guangxi Natural SF | Zheng M., et. al. 2017[11] |
| Impact of State Public Health Spending on Disease Incidence in the United States from 1980 to 2009. | deBeaumont Foundation | Verma R., et. al. 2017[12] |
| Death and the Media: Infectious Disease Reporting During the Health Transition. | NIA, NICHD | Costa DL., et. al. 2017[13] |
| Limits to Causal Inference with State-Space Reconstruction for Infectious Disease. |  | Cobey S., et. al. 2016[14] |
| Digital epidemiology reveals global childhood disease seasonality and the effects of immunization. |  | Bakker KM., et. al. 2016[15] |
| Online cross-validation-based ensemble learning. | NIAID, BMGF | Benkeser D., et. al. 2017[16] |
| Temporal Topic Modeling to Assess Associations between News Trends and Infectious Disease Outbreaks. | US Dept. of Interior, NIEHS | Ghosh S., et. al. 2017 [17] |
| Temperature Influences on Salmonella Infections across the Continental United States. |  | Uejio CK. 2017 [18] |
| **Conferences, pre-prints, theses, reports (11)** | | |
| Multiple peaks patterns of epidemic spreading in multi-layer networks. | China Nat. Natural SF, China Nat. Basic Res prog, Guangxi Natural SF | Zhen M., et. al., 2017[19] |
| A recursive point process model for infectious diseases. | NSF | Schoenberg F., et. al., 2017 [20] |
| The Historical Association between Tuberculosis and Diphtheria and a Modern Explanation. |  | Coleman S. year [21] |
| Projecting Climate-Related Disease Burden: A Guide for Health Departments. |  | Hess JJ., et. al., 2016[22] |
| Scalable Gaussian Processes for Characterizing Multidimensional Change Surfaces. | NSF | Herlands W., et. al., 2015[23] |
| FUNNEL: automatic mining of spatially coevolving epidemics. | JSPS KAKENHI, NSF | Matsubara Y., et. al., 2014[24] |
| On the predictability of infectious disease outbreaks. | Fondazoine Compagnia San Paolo, Fondation de l'Université d'Aix-Marseille | Scarpino SV., et. al., 2017[25] |
| H-Fuse: Efficient Fusion of Aggregated Historical Data. | NSF, IBM, Google | Liu Z., et. al., 2017[26] |
| Quantifying the impact of the measles vaccine by estimating the number of measles cases prevented for each state from 1964 to 2010 (*MPH Thesis*). |  | Sharbaugh M. 2016[27] |
| Efficient information integration system for temporal and spatial data (*PhD Thesis*). |  | Lee PJ. 2015[28] |
| Efficient Process Data Warehousing (*PhD Thesis*). |  | Hsu YF. 2015[29] |
| **Newspaper articles, blogs, websites (16)** | | |
| Battling Infectious Diseases in the 20th Century: The Impact of Vaccines (*Wall Street Journal*). |  | DeBold T., et. al., 2015[30] |
| The Vaccination Effect: 100 Million Cases of Contagious Disease Prevented (*New York Times*). |  | Lohr S. 2013[31] |
| The age of vaccines. |  | Scully T., et. al. 2014[32] |
| Worries Beyond Ebola: Infographic Shows What Else Is On America's Deadly Disease Watchlist (*Forbes*). |  | Bigman D. 2014[33] |
| Why Should You Get Immunized Against Measles? (*Infographic*) |  | Hydzik A., et. al., 2015[34] |
| SPEW VIEW (*Visualization*). |  | Gallagher S., et. al., 2016[35] |
| Contagious Diseases: Maps and Data Analysis (*Visualization*). |  | Lott B. 2017[36] |
| Disease history interactive map (*Software*). |  | LastMile 2015[37] |
| Project Tycho Utilities (*Software on Github*). |  | Gautier L. 2017[38] |
| Graph Database - Project Tycho (*Software*). |  | Ricker GG. 2014[39] |
| Data Stories - The impact of Vaccines on Measles (*Visualization*) |  | Vizlib Team. 2017[40] |
| Python wrapper for Project Tycho MMWR repository (*Software on Github*) |  | Rivers C. 2014 [41] |
| Gather the level 2 dataset from tycho project (*Software on Github*) |  | Zhang B. 2017 [42] |
| Interactive streamgraph visualization of measles cases over the 20th Century (*Visualization*) |  | Schurch N. 2015 [43] |
| Reproducing the WSJ Measles Vaccination Chart Using R (*Visualization*) |  | Lee M. 2017 [44] |
| Heatmaply: an R package for creating interactive cluster heatmaps for online publishing (*R-package*) |  | Galili T. 2017 [45] |

** Funders listed when acknowledged in published work*

Supplementary Table 4, Sources included in Project Tycho 2.0

| **Source name** | **Number of conditions** | **Number of countries** |
| --- | --- | --- |
| Cambodia Dengue Surveillance System | 1 | 1 |
| Laos Dengue Surveillance System | 1 | 1 |
| Malaysia Dengue Surveillance System | 1 | 1 |
| Pan American Health Organization Website | 2 | 50 |
| Philippines Sentinel Dengue Surveillance System | 1 | 1 |
| Singapore Dengue Surveillance System | 1 | 1 |
| Taiwan Notifiable Disease Surveillance System | 1 | 1 |
| Thailand Dengue Surveillance System | 1 | 1 |
| United States National Electronic Telecommunications System for Surveillance | 3 | 1 |
| United States Public Health Laboratory Information System | 3 | 1 |
| US Nationally Notifiable Disease Surveillance System | 92 | 1 |
| Vietnam Notifiable Disease Surveillance System | 1 | 1 |
| World Health Organization DengueNet Database | 3 | 89 |
| World Health Organization Southeast Asia Regional Office website | 1 | 10 |
| World Health Organization Western Pacific Regional Office Health Information Platform | 1 | 26 |
| World Health Organization Western Pacific Regional Office website | 2 | 37 |

Supplementary Table 5, Conditions and pathogens included in Project Tycho 2.0

| **Condition** | **SNOMED-CT** | **Pathogen** | **NCBI ID** | **Number of counts** |
| --- | --- | --- | --- | --- |
| Acquired immune deficiency syndrome | 62479008 | Human immunodeficiency virus | 12721 | 52316 |
| Active tuberculosis | 427099000 | Mycobacterium tuberculosis | 1773 | 11480 |
| Acute hepatitis C | 235866006 | Hepatitis C virus | 11103 | 671 |
| Acute nonparalytic poliomyelitis | 14535005 | Enterovirus C | 138950 | 6047 |
| Acute paralytic poliomyelitis | 240460008 | Enterovirus C | 138950 | 19462 |
| Acute poliomyelitis | 398102009 | Enterovirus C | 138950 | 137581 |
| Acute type A viral hepatitis | 25102003 | Human hepatitis A virus | 208726 | 41058 |
| Acute type B viral hepatitis | 76795007 | Hepatitis B virus | 10407 | 41277 |
| Amebic dysentery | 387754006 | Entamoeba histolytica | 5759 | 11708 |
| Anthrax | 409498004 | Bacillus anthracis | 1392 | 7100 |
| Aseptic meningitis | 301770000 | NA | NA | 41828 |
| Babesiosis | 21061004 | Babesiidae | 32594 | 3300 |
| Bacillary dysentery | 274081004 | Enterobacteriaceae | 543 | 11094 |
| Brucellosis | 75702008 | Brucella | 234 | 20002 |
| Campylobacteriosis | 86500004 | Campylobacter | 194 | 4486 |
| Chlamydia trachomatis infection | 240589008 | Chlamydia trachomatis | 813 | 7049 |
| Chlamydial infection | 105629000 | Chlamydia | 810 | 74097 |
| Cholera | 63650001 | Vibrio cholerae | 666 | 101 |
| Coccidioidomycosis | 60826002 | Coccidioides | 5500 | 10678 |
| Congenital rubella syndrome | 1857005 | Rubella virus | 11041 | 187 |
| Congenital syphilis | 35742006 | Treponema pallidum | 160 | 3927 |
| Cryptosporidiosis | 240370009 | Cryptosporidium | 5806 | 54082 |
| Dengue | 38362002 | Dengue virus | 12637 | 83892 |
| Dengue hemorrhagic fever | 20927009 | Dengue virus | 12637 | 10798 |
| Dengue without warning signs | 722862003 | Dengue virus | 12637 | 16331 |
| Diphtheria | 397428000 | Corynebacterium diphtheriae | 1717 | 370739 |
| Disease caused by West Nile virus | 417093003 | West Nile virus | 11082 | 611 |
| Disorder of nervous system caused by West Nile virus | 430397002 | West Nile virus | 11082 | 11758 |
| Dysentery | 111939009 | Multiple superkingdoms | 1 | 10444 |
| Encephalitis | 45170000 | Multiple superkingdoms | 1 | 6736 |
| Encephalitis lethargica | 186499007 | Multiple superkingdoms | 1 | 29711 |
| Giardiasis | 58265007 | Giardia | 5740 | 49482 |
| Gonorrhea | 15628003 | Neisseria gonorrhoeae | 485 | 163739 |
| Haemophilus influenzae infection | 91428005 | Haemophilus influenzae | 727 | 59450 |
| Haemophilus influenzae type b infection | 709410003 | NA | NA | 919 |
| Hepatitis non-A non-B | 186634008 | Viruses | 10239 | 34073 |
| Human anaplasmosis caused by Anaplasma phagocytophilum | 85708001 | Anaplasma phagocytophilum | 948 | 8464 |
| Human ehrlichiosis caused by Ehrlichia chaffeensis | 359747000 | Ehrlichia chaffeensis | 945 | 9784 |
| Infantile paralysis | 397928009 | Enterovirus C | 138950 | 29654 |
| Infection caused by Escherichia coli | 71057007 | Escherichia coli | 562 | 3581 |
| Infection caused by larvae of Trichinella | 709018004 | Trichinella | 6333 | 653 |
| Infection caused by non-cholerae vibrio | 398557001 | NA | NA | 765 |
| Infection caused by Shiga toxin producing Escherichia coli | 3.28291E+14 | NA | NA | 35127 |
| Infective encephalitis | 312215006 | Multiple superkingdoms | 1 | 46000 |
| Inflammatory disease of liver | 128241005 | Multiple superkingdoms | 1 | 26 |
| Influenza | 6142004 | unidentified influenza virus | 11309 | 221574 |
| Intestinal infection caused by Escherichia coli O157:H7 | 446328009 | Escherichia coli O157:H7 | 83334 | 30579 |
| Invasive drug resistant Streptococcus pneumoniae disease | 406618009 | Streptococcus pneumoniae | 1313 | 11686 |
| Invasive Group A beta-hemolytic streptococcal disease | 406614006 | Streptococcus sp. 'group A' | 36470 | 19270 |
| Invasive meningococcal disease | 707225006 | Neisseria meningitidis | 487 | 2063 |
| Invasive Streptococcus pneumoniae disease | 406617004 | Streptococcus pneumoniae | 1313 | 42208 |
| Legionella infection | 26726000 | Legionella | 445 | 67630 |
| Leprosy | 81004002 | Mycobacterium leprae | 1769 | 14217 |
| Listeriosis | 4241002 | Listeria monocytogenes | 1639 | 8599 |
| Lobar pneumonia | 278516003 | Multiple superkingdoms | 1 | 4209 |
| Lyme disease | 23502006 | Borrelia | 138 | 51254 |
| Malaria | 61462000 | Plasmodium | 5820 | 106082 |
| Measles | 14189004 | Measles virus | 11234 | 436932 |
| Meningitis | 7180009 | Multiple superkingdoms | 1 | 25812 |
| Meningococcal infectious disease | 23511006 | Neisseria meningitidis | 487 | 135789 |
| Meningococcal infectious disease | 23511006 | Neisseria meningitidis serogroup B | 491 | 660 |
| Meningococcal meningitis | 192644005 | Neisseria meningitidis | 487 | 126493 |
| Mumps | 36989005 | Mumps virus | 11161 | 165242 |
| Murine typhus | 25668000 | Rickettsiales | 766 | 2786 |
| Ornithosis | 75116005 | Chlamydia psittaci | 83554 | 824 |
| Pellagra | 418186002 | NA | NA | 29738 |
| Pertussis | 27836007 | Multiple superkingdoms | 1 | 297794 |
| Pneumonia | 233604007 | Multiple superkingdoms | 1 | 198788 |
| Post-infectious encephalitis | 192727001 | Multiple superkingdoms | 1 | 12918 |
| Primary encephalitis | 406573001 | Multiple superkingdoms | 1 | 26152 |
| Rocky Mountain spotted fever | 186772009 | Rickettsia rickettsii | 783 | 71024 |
| Rubella | 36653000 | Rubella virus | 11041 | 72676 |
| Salmonella infection | 302231008 | Salmonella | 590 | 71824 |
| Scarlet fever | 30242009 | Streptococcus sp. 'group A' | 36470 | 351882 |
| Shigellosis | 36188001 | Shigella | 620 | 63790 |
| Smallpox | 67924001 | Variola virus | 10255 | 260066 |
| Smallpox without rash | 74724001 | Variola virus | 10255 | 19 |
| Spotted fever group rickettsial disease | 186771002 | Rickettsia | 780 | 4742 |
| Streptococcal sore throat | 43878008 | Streptococcus sp. 'group A' | 36470 | 4642 |
| Syphilis | 76272004 | Treponema pallidum | 160 | 47 |
| Tetanus | 76902006 | Clostridium tetani | 1513 | 20251 |
| Toxic shock syndrome | 18504008 | Firmicutes | 1239 | 10981 |
| Tuberculosis | 56717001 | Mycobacterium tuberculosis | 1773 | 392715 |
| Tularemia | 19265001 | Francisella tularensis | 263 | 43851 |
| Typhoid and paratyphoid fevers | 186090001 | Salmonella | 590 | 58242 |
| Typhoid fever | 4834000 | Salmonella enterica subsp. enterica serovar Typhi | 90370 | 345041 |
| Typhus group rickettsial disease | 240613006 | Rickettsiales | 766 | 11447 |
| Varicella | 38907003 | Human herpesvirus 3 | 10335 | 97986 |
| Viral hepatitis | 3738000 | Viruses | 10239 | 27341 |
| Viral hepatitis type B | 66071002 | Hepatitis B virus | 10407 | 95518 |
| Viral hepatitis, type A | 40468003 | Human hepatitis A virus | 208726 | 80854 |
| West Nile fever without encephalitis | 397420007 | West Nile virus | 11082 | 9850 |
| Yellow fever | 16541001 | Yellow fever virus | 11089 | 68 |

Supplementary Table 6, DataCite attributes used in Project Tycho 2.0 metadata

| **Project Tycho dataset metadata attributes** | **Project Tycho dataset metadata values** |
| --- | --- |
| Identifier | dataset DOI |
| Title | Counts of <condition name> reported in <country ISO full name>: <earliest year>-<latest year> |
| Resource type general | Dataset |
| Resource type | Infectious disease incidence |
| Subject [1] | Disease Surveillance |
| Subject [1] value identifier | http://purl.obolibrary.org/obo/APOLLO_SV_00000545 |
| Subject [1] scheme | Apollo-SV |
| Subject [1] scheme identifier | <https://fairsharing.org/bsg-s002688> |
| Subject [2] | Disease Notification |
| Subject [2] value identifier | <http://purl.bioontology.org/ontology/MESH/D018563> |
| Subject [2] subject | Medical Subject Headings |
| Subject [2] scheme identifier | https://fairsharing.org/bsg-s000294 |
| Creator name identifier | creator ORCID |
| Creator name identifier scheme | Open Researcher and Contributor ID (ORCID) |
| Creator scheme identifier | https://fairsharing.org/FAIRsharing.nx58jg |
| Creator name | creator full name |
| Creator affiliation | affiliation name |
| Contributor type | type of contributor per DataCite specifications: http://schema.datacite.org/meta/kernel-4.0/include/datacite-contributorType-v4.xsd |
| Contributor name | full name of contributor, Last, first |
| Contributor name identifier | ORCID |
| Contributor name identifier scheme | Open Researcher and Contributor ID (ORCID) |
| Contributor scheme identifier | https://fairsharing.org/FAIRsharing.nx58jg |
| Contributor affiliation | affiliation of contributor |
| Publication year | 2018 |
| Date | date on which dataset was created, updated, or made available |
| Date type | created, updated, or available |
| Date type value identifier | https://schema.datacite.org/meta/kernel-4.0/include/datacite-dateType-v4.xsd |
| Language | en |
| GeoLocation place | <list of geographical subdivision levels included> in <country ISO full name> |
| Rights identifier | https://creativecommons.org/licenses/by-nc-sa/4.0/ |
| Rights | Attribution-NonCommercial-ShareAlike 4.0 International |
| Version | 2.00 |
| Format | CSV |
| Size | file size of dataset distribution (in MB) |
| Description | A description of the dataset |
| Description type | TechnicalInfo |
| Publisher | Project Tycho |
| Related identifier [1] | DOI of paper that describes methods to collect the dataset |
| Related identifier [1] relation | IsDocumentedBy, per http://schema.datacite.org/meta/kernel-4.0/include/datacite-relationType-v4.xsd |
| Related identifier [2] | DOI of paper that used the dataset |
| RelatedIdentifier [2] relation | IsCitedBy, per http://schema.datacite.org/meta/kernel-4.0/include/datacite-relationType-v4.xsd |
| Subject [1] | name of pathogen represented by the dataset |
| Subject [1] value identifier | URL of NCBI taxon ID represented by the dataset |
| Subject [1] scheme | NCBI Taxonomy |
| Subject [1] scheme identifier | <https://fairsharing.org/bsg-s000154> |
| Subject [2] | name of the condition represented by the dataset |
| Subject [2] value identifier | SNOMED CT page of condition |
| Subject [2] scheme | SNOMED-CT |
| Subject [2] scheme identifier | <https://fairsharing.org/bsg-s000098> |
| Subject [3] | NCBI Taxon ID of the pathogen represented by the dataset |
| Subject [3] value identifier | NCBI value page of taxon ID |
| Subject [3] scheme | NCBI Taxonomy |
| Subject [3] scheme identifier | <https://fairsharing.org/bsg-s000154> |
| Subject [4] | SNOMED-CT code of the condition represented by the dataset |
| Subject [4] value identifier | SNOMED CT page of condition |
| Subject [4] scheme | SNOMED-CT |
| Subject [4] scheme identifier | <https://fairsharing.org/bsg-s000098> |
| Subject [5] | ISO 3166 of the country represented by the dataset |
| Subject [5] value identifier | URL of ISO 3166 country page |
| Subject [5] scheme | ISO 3166 |
| Subject [5] scheme identifier | <https://www.iso.org/standard/63545.html> |
| Subject [6] | Case, if the dataset does not include fatalies |
| Subject [6] value identifier | <http://purl.bioontology.org/ontology/SNOMEDCT/398241000> |
| Subject [6] scheme | SNOMED-CT |
| Subject [6] scheme identifier | <https://fairsharing.org/bsg-s000098> |
| Subject [7] | Death, if the dataset includes fatalities |
| Subject [7] value identifier | <http://purl.bioontology.org/ontology/SNOMEDCT/419620001> |
| Subject [7] scheme | SNOMED-CT |
| Subject [7] scheme identifier | <https://fairsharing.org/bsg-s000098> |
| Subject [8] | <date earliest count>-<date last case count> |
| Subject [8] value identifier | URL of time interval page created by ontology |
| Subject [8] scheme | Time intervals ontology |
| Subject [8] scheme identifier | http://reference.data.gov.uk/def/intervals |
| Subject [9] | Cumulative incidence, if the dataset includes cumulative time series |
| Subject [9] value identifier | <http://purl.obolibrary.org/obo/EPO_0000061> |
| Subject [9] scheme | Epidemiology Ontology |
| Subject [9] scheme identifier | http://www.ontobee.org/ontology/EPO |
| Funding award identifier | URL of grant that funded the creation of the dataset |
| Funding award title | Title of grant that funded the creation of the dataset |
| Funding award number | Number of grant that funded the creation of the dataset |
| Funder identifier | Crossref Funder ID number |
| Funder identifier type | Crossref Funder ID |
| Funder name | Funder name |

Supplementary Table 7, DATS attributes used in Project Tycho 2.0 metadata

| **Project Tycho dataset metadata attribute** | **Project Tycho dataset metadata values** |
| --- | --- |
| Identifier | dataset DOI |
| Identifier source | Project Tycho |
| Title | Counts of <condition name> reported in <country ISO full name>: <earliest year>-<latest year> |
| Type of information | Infectious disease incidence |
| Type of information value identifier | <http://purl.obolibrary.org/obo/IDO_0000479> |
| Type of method [1] | Disease Surveillance |
| Type of method [1] value identifier | http://purl.o bolibrary.org/obo/APOLLO_SV_00000545 |
| Type of method [2] | Disease Notification |
| Type of method [2] value identifier | <http://purl.bioontology.org/ontology/MESH/D018563> |
| Creators identifier | ORCID of the dataset creator |
| Creators identifier source | https://fairsharing.org/FAIRsharing.nx58jg |
| Creators name | Name of creator |
| Creators email | Email of creator |
| Creators affiliation identifier | URL or creator affiliation |
| Creators affiliation name | Name of creator affiliation |
| Creator affiliation location identifier | URL of location in geonames |
| Creator affiliation location identifier source | http://www.geonames.org/ |
| Creator affiliation location name | Name of location |
| Creator affiliation location coordinates | Coordinates of location |
| Creator role | Creator role, per Scholarly Contributions and Roles Ontology |
| Creator role value identifier | URL of creator role in Scholarly Contributions and Roles Ontology |
| Date | Date on which dataset was created, updated, available |
| Date type | created, updated, or available |
| Date type value identifier | https://schema.datacite.org/meta/kernel-4.0/include/datacite-dateType-v4.xsd |
| Spatial coverage identifier | URL in ISO 3166 of country represented by the dataset |
| Spatial coverage identifier source | https://www.iso.org/standard/63545.html |
| Spatial coverage alternative identifier | URL in Geonames of country represented by the dataset |
| Spatial coverage alternative identifier source | http://www.geonames.org/ |
| Spatial coverage name | Name of country represented by the dataset |
| Spatial coverage description | <list of geographical subdivision levels included> in <country ISO full name> |
| License identifier | https://creativecommons.org/licenses/by-nc-sa/4.0/ |
| License name | Attribution-NonCommercial-ShareAlike 4.0 International |
| License version | 4.00 |
| License creator identifier | https://creativecommons.org/ |
| License creator name | Creative Commons |
| Distribution identifier | DOI of dataset |
| Distribution identifier source | Project Tycho |
| Distribution title | CSV file with counts of <condition name> reported in <country ISO full name>: <earliest year>-<latest year> |
| Distribution description | Downloadable CSV file of a Project Tycho dataset |
| Distribution date | Date on which dataset was created, updated, available |
| Distribution date type | created, updated, or available |
| Distribution date type identifier | https://schema.datacite.org/meta/kernel-4.0/include/datacite-dateType-v4.xsd |
| Identifier of repository that stores distribution | biodbcore-000995 |
| Source of identifier of repository | FAIRsharing |
| Alternative identifier of repository | [www.tycho.pitt.edu](http://www.tycho.pitt.edu/) |
| Name of repository that stores distribution | Project Tycho Repository |
| Description of repository that stores distribution | The Project Tycho Repository for Global Health Data aims to advance the availability and use of data for improving global health. A Project Tycho dataset includes case counts for a disease condition in a country. Data for Project Tycho datasets can come from various sources and have been pre-processed into the standard Project Tycho data format. |
| Date of repository that stores distribution | Date on which dataset distribution became available |
| Type of date of repository | Available |
| Source for type of date of repository | https://schema.datacite.org/meta/kernel-4.0/include/datacite-dateType-v4.xsd |
| Scope of repository that stores distribution | Infectious Disease Incidence |
| Value identifier for scope of repository | <http://purl.obolibrary.org/obo/IDO_0000479> |
| Type of repository that stores distribution | Primary Repository |
| License identifier of repository | https://creativecommons.org/licenses/by-nc-sa/4.0/ |
| License name of repository | Attribution-NonCommercial-ShareAlike 4.0 International |
| License version of repository | 4.00 |
| Identifier of license creator of repository | https://creativecommons.org/ |
| Name of license creator of repository | Creative Commons |
| Version of repository that stores distribution | 2.00 |
| Identifier of publisher of repository | http://www.pitt.edu/ |
| Name of publisher of repository | University of Pittsburgh |
| Location identifier of publisher of repository | <http://www.geonames.org/5206379/pittsburgh.html> |
| Source of location identifier of publisher | http://www.geonames.org/ |
| Name of location of publisher | Pittsburgh |
| Coordinates of location of publisher | 40.442906, -79.958548 |
| Access identifier of repository that stores distribution | [www.tycho.pitt.edu/data](http://www.tycho.pitt.edu/data) |
| Access landing page of repository | [http://www.tycho.pitt.edu/login](http://www.tycho.pitt.edu/login.php) |
| Type of access of repository | download |
| Authorization for access of repository | registration |
| Authentication for access of repository | simpleLogin |
| Identifier for standard that distribution of dataset conforms to | bsg-s000718 |
| Source of identifier for standard | FAIRsharing |
| Alternative identifier for standard | https://www.tycho.pitt.edu/dataformat/ProjectTychoPreCompiledDataFormat.pdf |
| Name of standard | Data format for Project Tycho 2.0 pre-compiled dataset CSV files |
| Type of standard | Format |
| Value identifier for type of standard | <http://edamontology.org/format_1915> |
| Description of standard | Specification of Project Tycho dataset file format and variable definitions |
| Version of standard | 1.00 |
| Dataset distribution format | CSV |
| Dataset distribution qualifier | isomorphic with dataset, zipped CSV, and Readme |
| Dataset distribution size | <file size of dataset distribution (in MB)> |
| Dataset distribution size unit | Megabyte |
| Value identifier of dataset distribution size unit | <http://purl.obolibrary.org/obo/UO_0000235> |
| Dataset distribution description | <textual description of the dataset distribution content> |
| Identifier of primary publication for dataset | DOI of dataset primary publication |
| Source of identifier for primary publication | Journal name of dataset primary publication |
| Title of primary publication | Title of dataset primary publication |
| Date of primary publication | Date of dataset primary publication |
| Type of date of primary publication | Available date |
| Value identifier for type of date | https://schema.datacite.org/meta/kernel-4.0/include/datacite-dateType-v4.xsd |
| Type of primary publication | Academic article |
| Venue of primary publication for dataset | Name of journal for dataset primary publication |
| Author identifier for primary publication | ORCID of author |
| Source of author identifier | https://fairsharing.org/FAIRsharing.nx58jg |
| Name of author for primary publication | Name of author |
| Email of author for primary publication | Email of author |
| Affiliation identifier for author of primary publication | URL of affiliation |
| Affiliation name for author of primary publication | Name of affiliation |
| Affiliation location identifier for author | Location identifier of affiliation in Geonames |
| Source of affiliation location identifier | http://www.geonames.org/ |
| Name of location for author | Name of location |
| Coordinates of location for author | Coordinates of location |
| Role of author for primary publication | Author role, per Scholarly Contributions and Roles Ontology |
| Value identifier for role of author | URL of author role, in Scholarly Contributions and Roles Ontology |
| Funding for primary publication | URL to grant that funded the primary publication |
| Funder identifier for primary publication | Crossref Funder ID for funder of primary publication |
| Source for funder identifier | https://www.crossref.org/services/funder-registry/ |
| Funder name for primary publication | Funder name |
| Identifier of dataset citation | DOI of paper citing the dataset |
| Title dataset citation | Title of paper citing the dataset |
| Date of paper citing dataset | Date of paper publication |
| Type of data for paper | Available date |
| Value identifier for type of date | https://schema.datacite.org/meta/kernel-4.0/include/datacite-dateType-v4.xsd |
| Type of citation | journal article, book, report |
| Venue of citation | Name of journal that published the paper citing the dataset |
| Subject [1] name | Name of the pathogen represented by the dataset |
| Subject [1] identifier | URL of NCBI Taxonomy page for pathogen |
| Subject [1] identifier source | https://fairsharing.org/bsg-s000154 |
| Subject [2] name | Name of the condition represented by the dataset |
| Subject [2] identifier | URL of SNOMED CT page of condition |
| Subject [2] identifier source | https://fairsharing.org/bsg-s000098 |
| Subject [3] name | NCBI Taxonomy ID of the pathogen represented by the dataset |
| Subject [3] identifier | URL of NCBI Taxonomy page for pathogen |
| Subject [3] identifier source | https://fairsharing.org/bsg-s000154 |
| Subject [4] name | SNOMED-CT code of condition |
| Subject [4] identifier | URL of SNOMED CT page of condition |
| Subject [5] name | ISO 3166 code of country |
| Subject [5] identifier | URL of ISO 3166 page of country |
| Subject [6] name | Case, if dataset does not include fatalities |
| Subject [6] identifier | <http://purl.bioontology.org/ontology/SNOMEDCT/398241000> |
| Subject [7] name | Death, if dataset includes fatalities |
| Subject [7] identifier | <http://purl.bioontology.org/ontology/SNOMEDCT/419620001> |
| Subject [8] name | <date earliest count>-<date last case count> |
| Subject [8] identifier | [URL](http://reference.data.gov.uk/id/gregorian-interval/1888-07-15T00:00:00/P1145288D) of Time Interval Ontology for time interval represented by the dataset |
| Subject [9] name | Cumulative incidence, if the dataset includes cumulative time series |
| Subject [9] identifier | <http://purl.obolibrary.org/obo/EPO_0000061> |
| Dataset funding | URL of grant that funded the creation of the dataset |
| Dataset funder identifier | Crossref ID of the funder that supported creation of the dataset |
| Funder identifier source | https://www.crossref.org/services/funder-registry/ |
| Dataset funder name | Name of funder |
| Identifier of researcher that received funding | ORCID of grant principal investigator |
| Source of identifier for researcher | https://fairsharing.org/FAIRsharing.nx58jg |
| Name of researcher that received funding | Name of grant principal investigator |
| Email of researcher that received funding | Email of grant principal investigator |
| Affiliation identifier of researcher that received funding | [www.pitt.edu](http://www.pitt.edu) |
| Name of affiliation | University of Pittsburgh |
| Location identifier of affiliation | <http://www.geonames.org/5206379/pittsburgh.html> |
| Source of location identifier | http://www.geonames.org/ |
| Name of location | Pittsburgh |
| Coordinates of location | 40.442906, -79.958548 |
| Role of researcher that received funding | Principal Investigator |
| Value identifier of role of researcher | <http://purl.org/spar/scoro/principal-investigator> |
| Dataset availability | available |
| Dataset refinement | data from public health agencies that has been reformatted and standardized |
| Dataset aggregation | instance of dataset |

Supplementary Table 8, Project Tycho standard data format for pre-compiled datasets

| **Attribute (* required)** | **Description** | **Value allowed** | **URL of vocabulary for attribute values** |
| --- | --- | --- | --- |
| ConditionName* | Name of reported condition as listed in SNOMED-CT | Any SNOMED-CT disorder name | doi:10.25504/fairsharing.d88s6e |
| ConditionSNOMED* | SNOMED-CT code for reported condition (https://biosharing.org/bsg-s000098) | Any SNOMED-CT disorder code | doi:10.25504/fairsharing.d88s6e |
| PathogenName | Organism name for pathogen causing reported condition | Any organism name in the NCBI Taxonomy, or NA | doi:10.25504/fairsharing.fj07xj |
| PathogenTaxonID | NCBI Taxonomy identifier for pathogen causing reported condition | Any NCBI Taxonomy ID, or NA | doi:10.25504/fairsharing.fj07xj |
| Fatalities* | Counts of reported condition represent fatalities | 1, 0 | No external standard |
| CountryName* | ISO 3166 English Short Name of country for which a count was reported | Any ISO 3166 country English Short Name (all capitals), as of 07-24-2017 | https://www.iso.org/standard/63545.html |
| CountryISO | ISO 3166 2-letter code for country | Any 2-letter ISO 3166 country code, as of 07-24-2017 | https://www.iso.org/standard/63545.html |
| Admin1Name | ISO 3166 Name of first administrative subdivision for which a count was reported | Any ISO 3166 fist administrative subdivision name (all capitals), as of 07-24-2017, or NA | https://www.iso.org/standard/63545.html |
| Admin1ISO | ISO 3166-2 code for first administrative subdivision | Any ISO 3166-2 code for first administrative subdivision, as of 07-24-2017, or NA | https://www.iso.org/standard/63545.html |
| Admin2Name | Place name from Geonames of second-order administrative division for which a count was reported | Any place name from Geonames of second-order administrative division (all capitals), or NA | http://www.geonames.org/ |
| CityName | Name of Populated place for which a count was reported, from Geonames | Any name from Geonames for (part of) a populated place, seat, capital, or similar feature class (all capitals), or NA | http://www.geonames.org/ |
| PeriodStartDate* | Start date of time interval for which a count was reported, in ISO 8601 format | Any date in ISO 8601 format | https://www.iso.org/iso-8601-date-and-time-format.html |
| PeriodEndDate* | End date of time interval for which a count was reported, in ISO 8601 format | Any date in ISO 8601 format | https://www.iso.org/iso-8601-date-and-time-format.html |
| PartOfCumulativeCountSeries* | Count is part of a series of cumulative counts (instead of being part of a series of fixed time interval counts) | 0,1 | No external standard |
| AgeRange* | Age range for which a count was reported (in years) | Any age range between 0 and 130 with age values separated by “-” as in: “0-130” | No external standard |
| Subpopulation* | A subgroup of the population for which a count was reported | “Civilian”, “Military”, “None specified” | No external standard |
| PlaceOfAquisition | Place where reported condition was acquired: “Domestic” (i.e., in the country for which a case count was reported) or “Abroad” (i.e., not in the country for which the case count was reported) | “Domestic”, “Abroad”, or NA | No external standard |
| DiagnosisCertainty | Qualifier for certainty of diagnosis for a count condition | “Definite”, “Equivocal”,  “Possible diagnosis”,  “Probable diagnosis”, or NA | doi:10.25504/fairsharing.d88s6e |
| SourceName* | Name of the source (system, database, institution) from which counts were obtained by the Project Tycho team | E.g., “Nationally Notifiable Disease Surveillance System”, “World Health Organization DengueNet Database” | No external standard |
| CountValue* | The count value | Any whole number | No external standard |


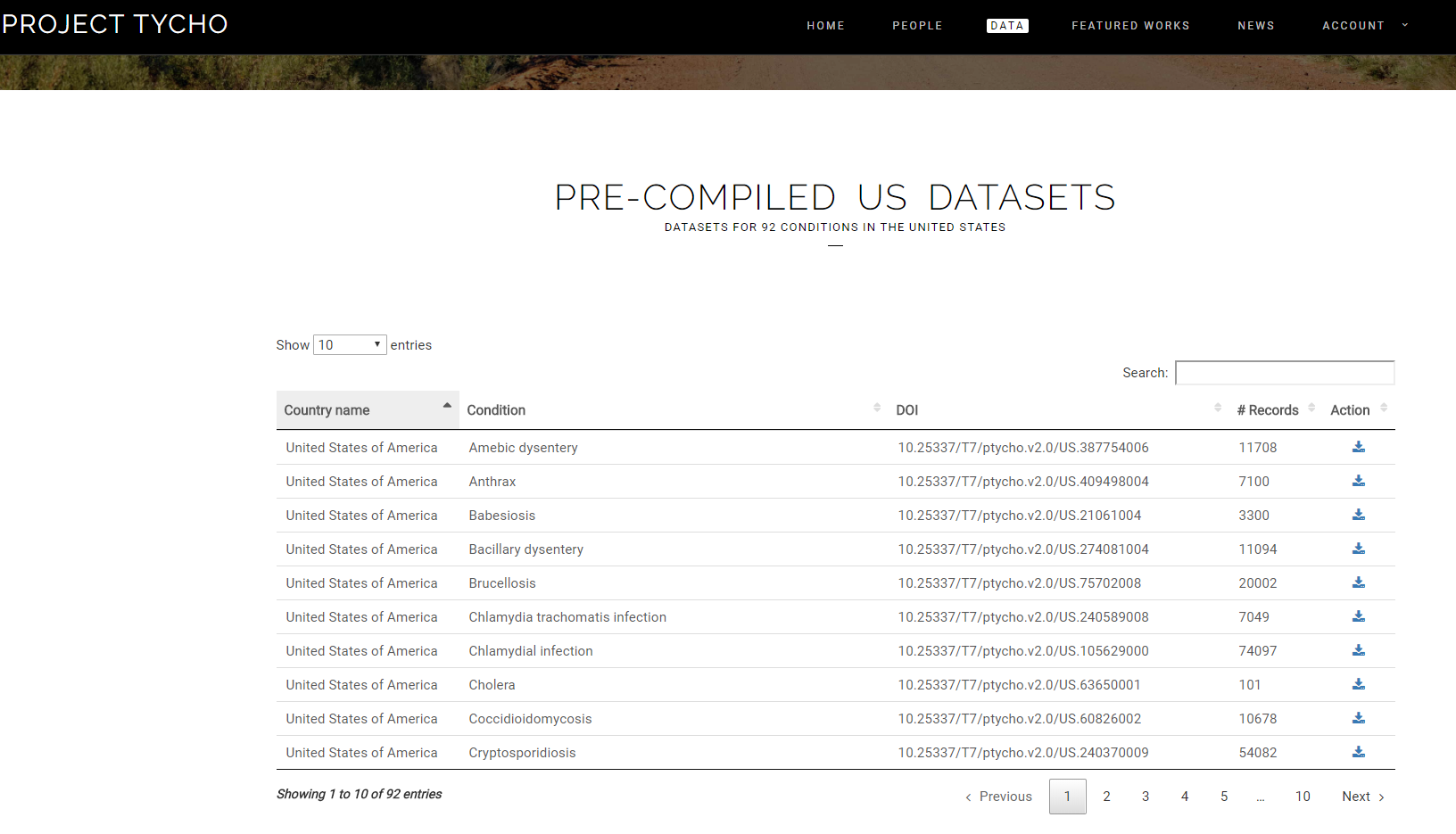


B

A


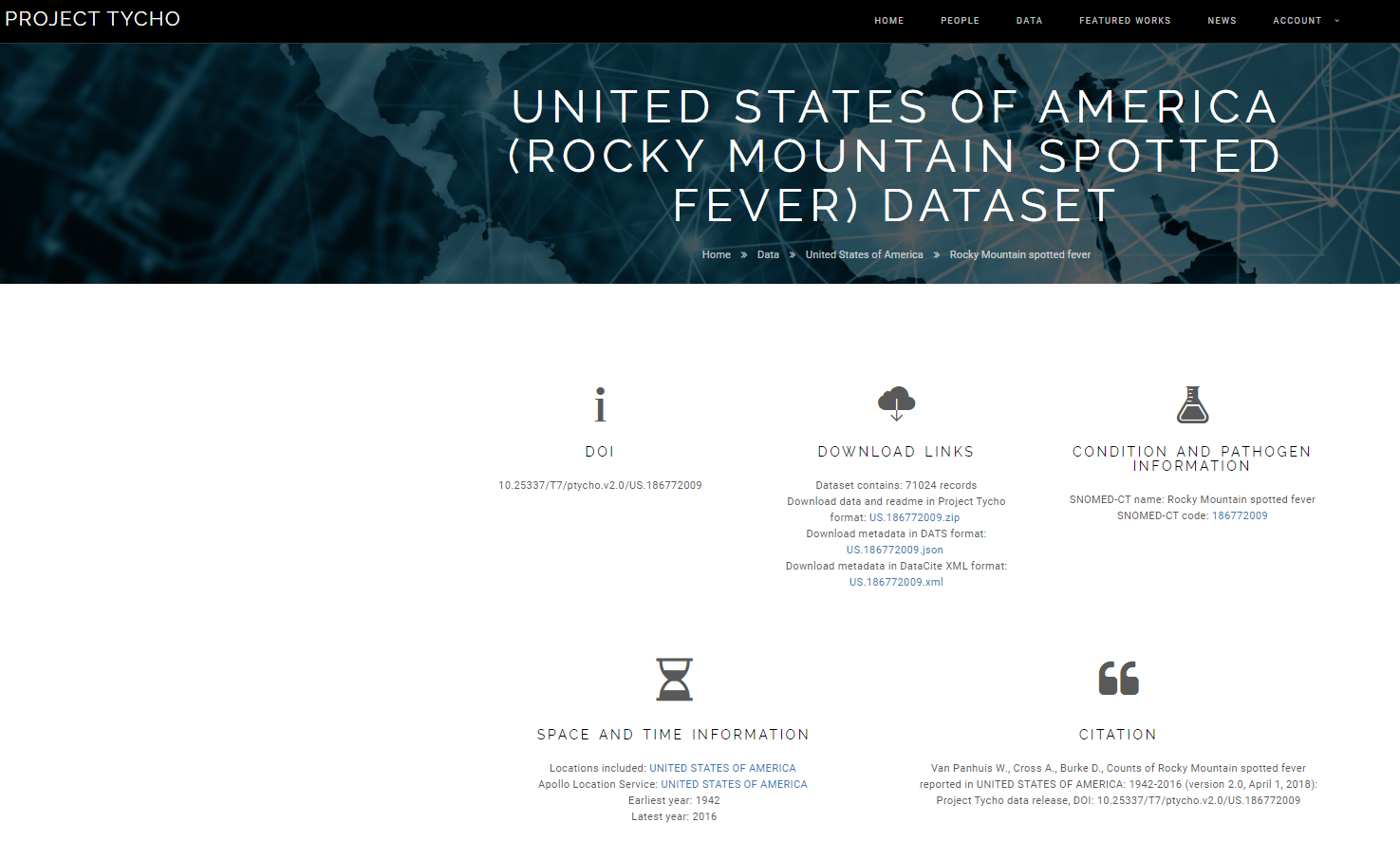


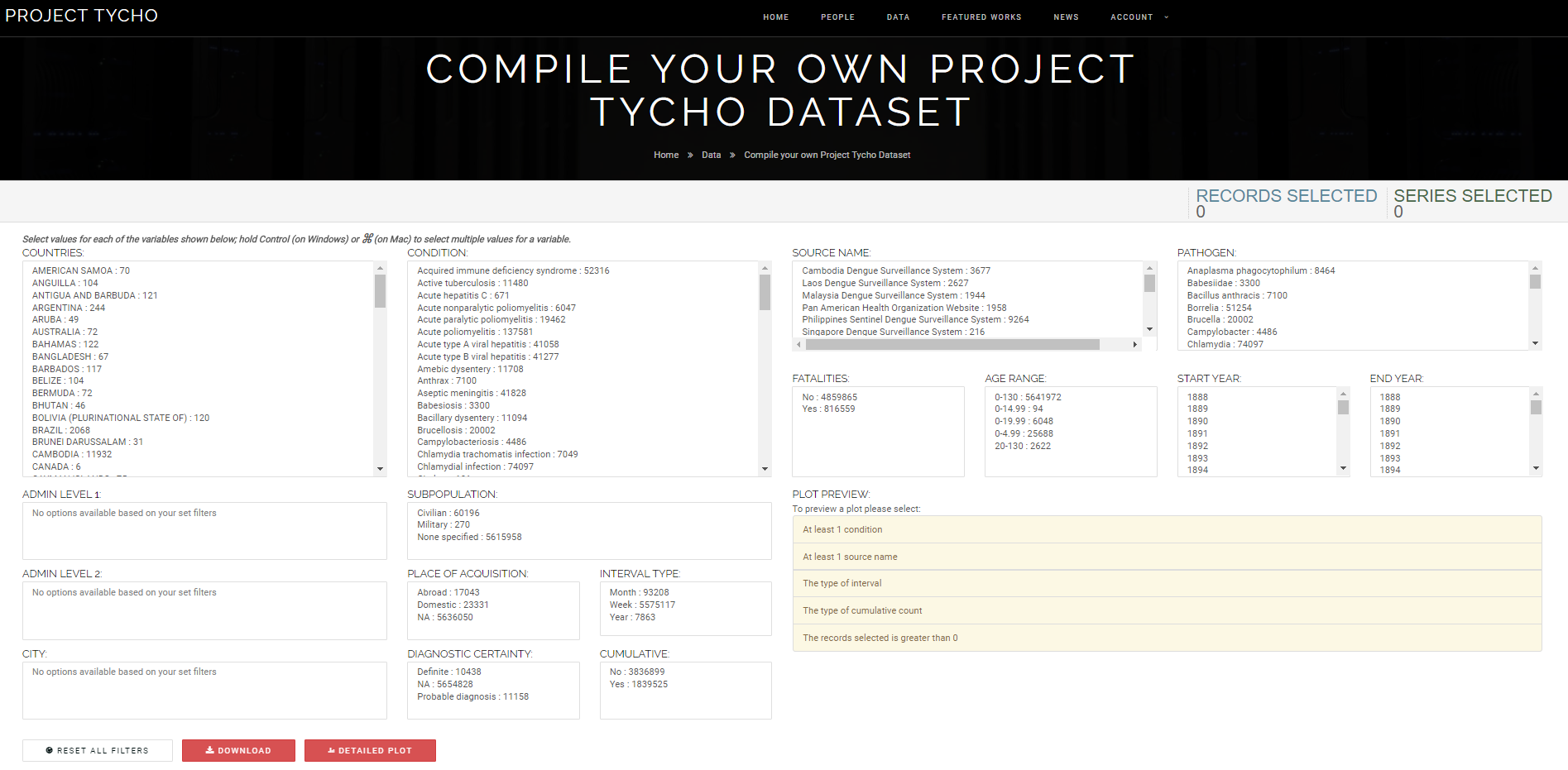


C

**Supplementary Figure 1, Screenshots of Project Tycho v2 data retrieval pages. (A)** Listing of Project Tycho v2 pre-complied datasets; **(B)** the landing page of the pre-compiled dataset with counts of Rocky Mountain Spotted Fever in the United States; and **(C)** the graphical user interface for compiling a custom datasets

**REFERENCES**

1 Coleman S. The historical association between measles and pertussis: A case of immune suppression? *SAGE Open Med* 2015;**3**:205031211562131. doi:10.1177/2050312115621315

2 Coleman S. The association between varicella (chickenpox) and group A streptococcus infections in historical perspective. *SAGE Open Med* 2016;**4**:205031211665890. doi:10.1177/2050312116658909

3 Dalziel BD, Bjørnstad ON, van Panhuis WG, *et al.* Persistent Chaos of Measles Epidemics in the Prevaccination United States Caused by a Small Change in Seasonal Transmission Patterns. *PLOS Comput Biol* 2016;**12**:e1004655. doi:10.1371/journal.pcbi.1004655

4 Shrestha S, Foxman B, Berus J, *et al.* The role of influenza in the epidemiology of pneumonia. *Sci Rep* 2015;**5**:15314. doi:10.1038/srep15314

5 Althouse BM, Scarpino S V. Asymptomatic transmission and the resurgence of Bordetella pertussis. *BMC Med* 2015;**13**:146. doi:10.1186/s12916-015-0382-8

6 Mina MJ, Metcalf CJE, de Swart RL, *et al.* Long-term measles-induced immunomodulation increases overall childhood infectious disease mortality. *Science (80- )* 2015;**348**:694–9. doi:10.1126/science.aaa3662

7 Magpantay FMG, Rohani P. Dynamics of Pertussis Transmission in the United States. *Am J Epidemiol* 2015;:kwv024-. doi:10.1093/aje/kwv024

8 Martinez-Bakker M, King AA, Rohani P. Unraveling the Transmission Ecology of Polio. *PLOS Biol* 2015;**13**:e1002172. doi:10.1371/journal.pbio.1002172

9 Scarpino S V., Allard A, Hébert-Dufresne L. The effect of a prudent adaptive behaviour on disease transmission. *Nat Phys* 2016;**12**:1042–6. doi:10.1038/nphys3832

10 Costa DL, Kahn ME. Declining Mortality Inequality within Cities during the Health Transition. *Am Econ Rev* 2015;**105**:564–9. doi:10.1257/aer.p20151070

11 Zheng M, Zhao M, Min B, *et al.* Synchronized and mixed outbreaks of coupled recurrent epidemics. *Sci Rep* 2017;**7**:2424. doi:10.1038/s41598-017-02661-9

12 Verma R, Clark S, Leider J, *et al.* Impact of State Public Health Spending on Disease Incidence in the United States from 1980 to 2009. *Health Serv Res* 2017;**52**:176–90. doi:10.1111/1475-6773.12480

13 Costa DL, Kahn ME. Death and the Media: Infectious Disease Reporting During the Health Transition. *Economica* 2017;**84**:393–416. doi:10.1111/ecca.12227

14 Cobey S, Baskerville EB, Abarbanel H, *et al.* Limits to Causal Inference with State-Space Reconstruction for Infectious Disease. *PLoS One* 2016;**11**:e0169050. doi:10.1371/journal.pone.0169050

15 Bakker KM, Martinez-Bakker ME, Helm B, *et al.* Digital epidemiology reveals global childhood disease seasonality and the effects of immunization. *Proc Natl Acad Sci U S A* 2016;**113**:6689–94. doi:10.1073/pnas.1523941113

16 Benkeser D, Ju C, Lendle S, *et al.* Online cross-validation-based ensemble learning. *Stat Med* Published Online First: 2017. doi:10.1002/sim.7320

17 Ghosh S, Chakraborty P, Nsoesie EO, *et al.* Temporal Topic Modeling to Assess Associations between News Trends and Infectious Disease Outbreaks. *Sci Rep* 2017;**7**:40841. doi:10.1038/srep40841

18 Uejio CK. Temperature Influences on Salmonella Infections across the Continental United States. *Ann Am Assoc Geogr* 2017;**107**:751–64. doi:10.1080/24694452.2016.1261681

19 Zheng M, Wang W, Tang M, *et al.* Multiple peaks patterns of epidemic spreading in multi-layer networks. Published Online First: 2017.https://arxiv.org/pdf/1706.05780.pdf (accessed 11 Aug 2017).

20 Schoenberg F, Hoffmann M, Harrigan R. A recursive point process model for infectious diseases. Published Online First: 23 March 2017.https://arxiv.org/pdf/1703.08202.pdf (accessed 11 Aug 2017).

21 Coleman S. The Historical Association between Tuberculosis and Diphtheria and a Modern Explanation. 2017.https://papers.ssrn.com/sol3/papers.cfm?abstract_id=2985642 (accessed 11 Aug 2017).

22 Hess JJ, Saha S, Schramm PJ, *et al.* Projecting Climate-Related Disease Burden: A Guide for Health Departments. Atlanta: 2016. http://www.cdc.gov/climateandhealth/pubs/projectingclimaterelateddiseaseburden1_508.pdf (accessed 11 Aug 2017).

23 Herlands W, Wilson A, Nickisch H, *et al.* Scalable Gaussian Processes for Characterizing Multidimensional Change Surfaces. Published Online First: 13 November 2015.http://arxiv.org/abs/1511.04408

24 Matsubara Y, Sakurai Y, van Panhuis WG, *et al.* FUNNEL: automatic mining of spatially coevolving epidemics. In: *Proceedings of the 20th ACM SIGKDD international conference on Knowledge discovery and data mining - KDD ’14*. New York, New York, USA: : ACM Press 2014. 105–14. doi:10.1145/2623330.2623624

25 Scarpino S V., Petri G. On the predictability of infectious disease outbreaks. Published Online First: 21 March 2017.http://arxiv.org/abs/1703.07317

26 Liu Z, Song HA, Zadorozhny V, *et al.* H-Fuse: Efficient Fusion of Aggregated Historical Data. In: *Proceedings of the 2017 SIAM International Conference on Data Mining*. Philadelphia, PA: : Society for Industrial and Applied Mathematics 2017. 786–94. doi:10.1137/1.9781611974973.88

27 Sharbaugh M. *Quantifying the impact of the measles vaccine by estimating the number of measles cases prevented for each state from 1964 to 2010*. 2015.http://d-scholarship.pitt.edu/26393/ (accessed 11 Aug 2017).

28 Lee P-J. *EFFICIENT INFORMATION INTEGRATION SYSTEM FOR TEMPORAL AND SPATIAL DATA*. 2015.http://d-scholarship.pitt.edu/25131/ (accessed 11 Aug 2017).

29 Hsu Y-F. *Efficient Process Data Warehousing*. 2015.https://search.proquest.com/pqdtglobal/docview/1777531131/abstract/6C66814C3D444C5DPQ/5?accountid=14709 (accessed 11 Aug 2017).

30 DeBold T, Friedman D. Battling Infectious Diseases in the 20th Century: The Impact of Vaccines. Wall Str. J. 2015.http://graphics.wsj.com/infectious-diseases-and-vaccines/

31 Lohr S. The Vaccination Effect: 100 Million Cases of Contagious Disease Prevented. New York Times. 2013.https://bits.blogs.nytimes.com/2013/11/27/the-vaccination-effect-100-million-cases-of-contagious-disease-prevented/?_r=0%0A

32 Scully T. The age of vaccines. *Nature* 2014;**507**:S2–3. doi:10.1038/507S2a

33 Bigman D. Worries Beyond Ebola: Infographic Shows What Else Is On America’s Deadly Disease Watchlist. *Forbes Mag* 2014.https://www.forbes.com/sites/danbigman/2014/10/15/beyond-ebola-what-else-is-on-americas-deadly-disease-watchlist/#f7293967b7ef%0A

34 Hydzik A, Davison N. Why Should You Get Immunized Against Measles? Insid. Life Chang. Med. 2015.http://insideupmc.upmc.com/why-should-you-get-immunized-against-measles/?sf36335511=1%0A (accessed 8 Nov 2017).

35 Gallagher S, Richardson L, Ventura S, *et al.* SPEW VIEW. 2016.http://stat.cmu.edu:3838/sgallagh/hackathon/ (accessed 11 Aug 2017).

36 Lott B. Contagious Diseases: Maps and Data Analysis | Kaggle. 2017.https://www.kaggle.com/benjaminlott/contagious-diseases-maps-and-data-analysis (accessed 11 Aug 2017).

37 LastMile, Code.7370, Calpia. Disease history interactive map. 2015.http://www.tycho.pitt.edu/resources/lastmile/

38 Gautier L. Project Tycho Utilities. Github Repos. 2017.https://github.com/lgautier/project-tycho-utilities (accessed 8 Nov 2017).

39 Ricker GG. Graph Database - Project Tycho. Pers. blog. 2014.https://rickerg.com/2014/04/07/graph-database-project-tycho/ (accessed 8 Nov 2017).

40 Vizlib Team. Data Stories - The impact of Vaccines on Measles. Vizlib | Blog. 2017.https://www.vizlib.com/impactofvaccinesonmeasles/ (accessed 6 Apr 2018).

41 Rivers C. Python wrapper for Project Tycho MMWR repository. Github Repos. 2014.https://github.com/cmrivers/pycho (accessed 7 Apr 2018).

42 Zhang B. Gather the level 2 dataset from tycho project. Github Repos. 2017.https://github.com/Spatial-R/IDDC (accessed 6 Apr 2018).

43 Schurch N. Interactive streamgraph visualization of measles cases over the 20th Century. Figshare. 2015.https://figshare.com/articles/Interactive_streamgraph_visualization_of_measles_cases_over_the_20th_Century/1379899 (accessed 6 Apr 2018).

44 Lee M. Reproducing the WSJ Measles Vaccination Chart Using R. Pers. blog. 2017.https://www.mikelee.co/posts/2017-06-28-wsj-measles-vaccination-chart/ (accessed 6 Apr 2018).

45 Galili T. heatmaply: an R package for creating interactive cluster heatmaps for online publishing. R-bloggers. 2017.https://www.r-bloggers.com/heatmaply-an-r-package-for-creating-interactive-cluster-heatmaps-for-online-publishing/ (accessed 6 Apr 2018).
